# High‐Throughput 3D Glioblastoma Model in Glycosaminoglycan Hydrogels for Personalized Therapeutic Screening

**DOI:** 10.1002/mabi.202500394

**Published:** 2026-01-14

**Authors:** Rajvinder Kaur Trautmann, Nicholas Dennison, Kathleen McCortney, Solveig Klier, Mehmet Ilyas Cosacak, Carsten Werner, Goktug Akyoldas, Craig M. Horbinski, Uwe Freudenberg, Caghan Kizil

**Affiliations:** ^1^ Neuron‐D GmbH Dresden Germany; ^2^ Leibniz Institute of Polymer Research Dresden (IPF) Dresden Germany; ^3^ Nervous System Tumor Bank Department of Neurological Surgery Feinberg School of Medicine Northwestern University Chicago Illinois USA; ^4^ German Center for Neurodegenerative Diseases (DZNE) Dresden Germany; ^5^ Koc University Hospital İstanbul Türkiye; ^6^ Departments of Pathology and Neurosurgery Feinberg School of Medicine Northwestern University Chicago Illinois USA; ^7^ Department of Neurology Vagelos College of Physicians and Surgeons Columbia University Irving Medical Center New York New York USA

**Keywords:** bioengineered tumor microenvironment, Glioblastoma, high‐throughput screening, patient‐derived cells, personalized medicine, starPEG‐heparin hydrogel

## Abstract

Glioblastoma (GBM) is a devastating brain tumor with limited treatment success, partly because *in vitro* models poorly mimic *in vivo* complexity. This study introduces a high‐throughput 3D culture platform utilizing modular starPEG–glycosaminoglycan (GAG) hydrogels that enable independent control of extracellular matrix (ECM) cues: stiffness, cytokine affinity, matrix metalloproteinase‐responsive remodeling, and cell adhesiveness via integrin‐binding RGD peptides. This platform supports encapsulation of patient‐derived GBM cells, recreates physiologically relevant tumor microenvironments in 384‐well plates, and enables automated drug testing on primary cells. Transcriptomic analyses show that 3D cultures recapitulate primary and recurrent GBM programs‐ including hypoxia‐, immune‐, and ECM‐regulatory pathways driving growth, invasion, and resistance, without externally imposed hypoxia. The platform's versatility extends to drug screening, where single and combinatorial treatments produce reproducible cytoskeletal and transcriptomic responses. Notably, the system revealed dose‐dependent reductions in invasive filaments and spheroid architecture with 5‐fluorouracil/uridine and carmustine, demonstrating its potential for optimizing combinatorial therapies. This 3D model surpasses 2D cultures, capturing tumor‐specific molecular programs and offering a robust tool for translational research. Despite lacking vascular or immune components, its tunability, scalability, and clinical relevance make it a strong basis for advanced co‐cultures. By delivering reliable, individualized therapeutic data within a short timeframe, this model holds transformative potential for personalized GBM treatment.

## Graphical abstract

Kaur–Trautmann et al. present a high‐throughput, chemically defined 3D glioblastoma model based on glycosaminoglycan hydrogels that allow orthogonal control of stiffness, adhesion, and degradability while standardizing soluble‐factor presentation. The platform supports automated imaging and transcriptomic profiling, enabling patient‐specific drug screening and revealing metabolic and microenvironmental adaptations relevant to personalized therapy development.

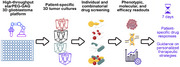

## 1 Introduction

Glioblastoma (GBM) is one of the most aggressive and lethal brain tumors, characterized by rapid growth, extensive invasion into surrounding brain tissue, and profound heterogeneity at both cellular and molecular levels [1, 2, 3, 4]. Despite advances in surgery, radiotherapy, and chemotherapy, the prognosis for GBM patients remains poor, with a median survival of approximately 15 months following diagnosis [5, 6, 7]. This poor prognosis is partly due to the lack of physiologically relevant preclinical models capable of recapitulating the complexity of the GBM tumor microenvironment (TME) [8, 9]. The TME plays a pivotal role in driving GBM progression, therapeutic resistance, immune evasion, and immunosuppression [10]. It consists of a dynamic interplay among tumor cells, immune cells, vascular components, and the extracellular matrix (ECM) [11, 12, 13]. Equally important, soluble factors, including cytokines and growth factors, play crucial roles in the dynamic regulation of cell behavior, therapeutic responses, and tumor progression within the TME [14, 15]. Key cytokines, such as VEGF and TGF‐β, are known to promote angiogenesis and therapy resistance in GBM, making their controlled presentation *in vitro* essential for realistic drug testing [15]. Therefore, both the physical and biochemical aspects of the TME must be precisely managed in vitro to understand and control GBM progression more effectively [16].

Over the past decade, 3D GBM models have surpassed 2D monolayers in terms of physiological relevance [17, 18, 19, 20]. Patient‐derived spheroids and organoids retain intratumoral heterogeneity and microenvironmental gradients (e.g., hypoxia), whereas ECM‐defined hydrogels‐ including hyaluronan, collagen, gelatin, PEG, and composite matrices‐ permit precise control of matrix stiffness and ligand presentation [21, 22, 23, 24, 25]. In parallel, microphysiological BBB‐on‐chip devices introduce perfusion and barrier transport [26]. Nevertheless, each modality presents distinct limitations: organoids and chips pose challenges in terms of standardization and scalability for systematic screening, and often lack well‐defined ECM components, which are essential for accurately evaluating drug efficacy; many hydrogel systems modulate mechanics but lack explicit control of soluble factor presentation or cannot orthogonally decouple stiffness, ligand density, and degradability; and automation‐compatible, plate‐based formats that retain a chemically defined ECM remain uncommon [17, 19, 21, 27]. Thus, despite the consensus that 3D outperforms 2D for GBM, the remaining gap is a platform that couples biological fidelity with standardization and scale. Collectively, these limitations motivate the development of a platform that unites orthogonal control of ECM‐cues with standardized GAG‐mediated soluble factor presentation, enabling automation‐ready, low‐volume, multiplexed assays on patient‐derived GBM cells within clinically relevant timescales [28, 29, 30].

To meet these requirements, we designed a matrix to reproduce the key features of the brain ECM. Brain tissue is rich in glycosaminoglycans (GAGs) and sulfated proteoglycans, which shape the biophysical milieu and critically regulate signaling by reversible administration of cytokines, chemokines, and growth factors [30, 31, 32]. While hyaluronan is abundant yet unsulfated and therefore binds many growth factors only weakly [33, 34, 35], sulfated GAGs support high‐affinity, reversible interactions that localize and temporally integrate soluble factors implicated in GBM progression [30, 36, 37]. Guided by this biology, we employed starPEG–GAG hydrogels as chemically defined scaffolds that afford orthogonal control of stiffness (via polymer content/crosslinking density), integrin‐mediated adhesion (e.g., RGD), and MMP‐sensitive degradability, while the GAG component standardizes soluble factor presentation through reversible sequestration [21, 23, 27, 36].

Here, we present a high‐throughput 3D starPEG‐GAG‐ hydrogel system that links defined matrix properties to plate‐based, automation‐compatible workflows. The platform (i) independently tunes stiffness, adhesion, and degradability; (ii) leverages GAG‐mediated, reversible growth factor sequestration to standardize soluble signaling; and (iii) supports low‐volume culture with multiplexed readouts—automated 3D imaging of architecture plus transcriptomic profiling—delivering decision‐relevant responses within ∼7 days. We demonstrate reproducible and scalable analyses with an established cell line (LN229) and patient‐derived GBM cells (NU01520 and KUGBM8), positioning this defined, automation‐ready approach for personalized therapeutic screening. Our findings highlight the potential of our platform to bridge the gap between preclinical models and clinical applications, providing a scalable and customizable tool for advancing GBM research. This approach could enable the rapid and reliable discovery of patient‐specific therapeutic responses, thereby fostering personalized medicine.

## 2 Results and Discussion

GBM remains an unmet need, in part because standard *in vitro* models lack an ECM–mimetic microenvironment that can be scaled for drug screening. Here, we present a fully automated 384‐well platform built on matrix‐metalloproteinase‐responsive starPEG‐GAG hydrogels, whose independently tunable stiffness (≈1 kPa), RGD‐peptide density, and degradable crosslinks recapitulate key brain ECM features. We demonstrate that (1) these material cues prime LN229 and patient‐derived GBM cells toward invasive, filamentous phenotypes; (2) quantitative 3D readouts, such as total filament volume and spheroid metrics, capture dose‐dependent responses to monotherapy and reveal enhanced effects under combination treatments; and (3) bulk transcriptomes of 3D cultures enrich for pathways linked to ECM remodeling, invasion, and adaptive resistance, which are attenuated in 2D monolayers. Together, these findings establish a biomaterials‐centric route to model GBM‐relevant behaviors in a high‐throughput format, laying the groundwork for future co‐culture and perfused BBB‐on‐chip extensions.

### 2.1 Automated Preparation and Analysis of High‐Throughput 3D GBM Cultures in Cell‐Instructive starPEG‐GAG Hydrogels (3D‐HT‐GBM)

The overarching goal of our study was to establish a robust platform for high‐throughput investigation of GBM cell behavior, morphological adaptations, and treatment responses, with an emphasis on patient‐specific contexts. To address this, we developed a high‐throughput 3D culture system utilizing cell‐instructive starPEG‐GAG hydrogels that use heparin as a highly sulfated GAG‐building block integrated within automated fluid handling and microscopy platforms. We started by evaluating the cellular morphology of LN229 and patient‐derived GBM cells (NU01520) within the cell‐instructive hydrogels.

Hydrogels were crosslinked through a rapid, *in situ*, bio‐orthogonal Michael‐type crosslinking reaction between thiol‐terminated starPEG‐MMP (PEG‐SH) and maleimide‐ functionalized heparin (HM6), forming a homogeneous hydrogel matrix. RGDSP peptides were covalently conjugated to excess heparin maleimide groups before crosslinking to enable integrin‐mediated cell adhesion [36, 38, 39]. Hydrogel stiffness (crosslinking density) was set by the thiol:maleimide functional‐equivalent ratio (γ) together with total polymer solids content; unless stated otherwise, γ is calculated excluding pre‐conjugated RGD, allowing adhesion to be tuned independently at fixed crosslinking. Cell‐responsive remodeling was enabled independently of the crosslinking density via the presence of an MMP‐cleavable linker on all arms of the starshaped PEG, and swelling was constrained by keeping the polymer solid content within a narrow range. In parallel, we empirically optimized the cell seeding density, medium composition, and growth factor supplementation (at fixed hydrogel matrix settings) to elicit robust GBM architectures. The screening formulation used throughout was γ = 1.50 and 2.8% (w/v) total polymer solid content. Rheological measurements confirmed that γ = 1.50 yielded a storage modulus of ∼1 kPa, closely matching brain tissue stiffness; this condition consistently produced the most physiologically relevant results and was used as the standard across lines. Under screening conditions, 3D cultures reproducibly exhibited physiologically relevant GBM architectures, including coexisting compact spheroids and invasive, stellate F‐actin–rich networks. Morphologies were quantified from whole‐well, stitched confocal z‐stacks (70–320 µm, 51 z‐planes, total depth ∼250 µm) in Imaris; this whole‐well strategy minimizes field‐selection/edge effects and yields low well‐to‐well variance in the 384‐well format; only minor adjustments (typically medium or seeding density) were required when introducing new patient‐derived lines.

**FIGURE 1:**
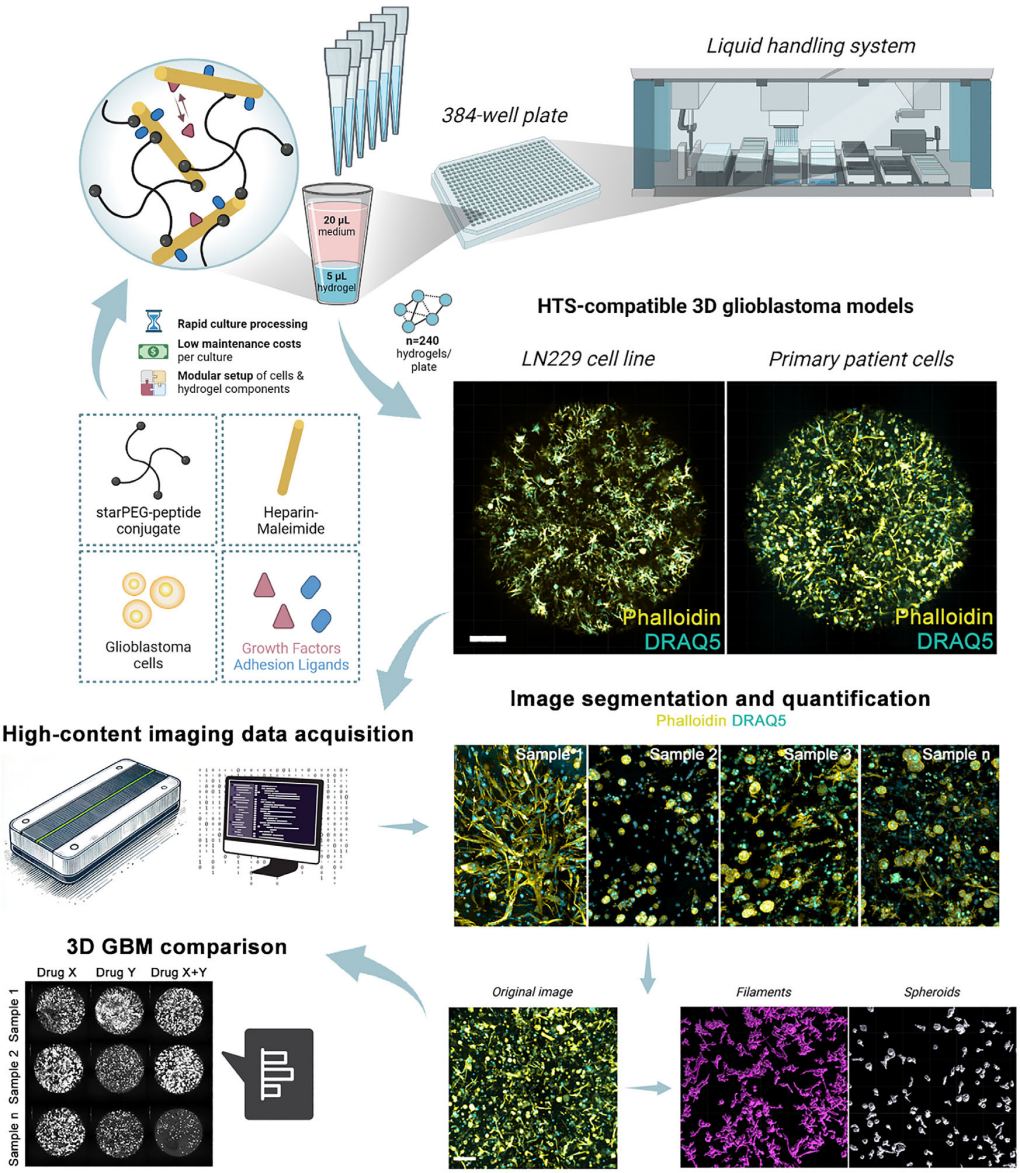
Schematic overview of automated generation of parallel 3D hydrogel cultures, high‐content imaging, and image segmentation in GBM model. The hydrogel fabrication involved the preparation of two distinct stock solutions. The starPEG‐peptide conjugate and the heparin‐maleimide/RGDSP peptide/embedded GBM cell mixture were automatically transferred and mixed in a 96‐well plate with V‐shaped wells, before being dispensed into a low‐volume 384‐well plate, with a volume of 5 µL per well, using an automated liquid handling system, such as the Janus or Tecan. Following a 7‐day culture period, the cellular distribution and morphology within the 3D hydrogels were examined by fixing the cells and staining for filamentous actin (F‐actin) using phalloidin‐FITC (yellow), which specifically binds to F‐actin to visualize cytoskeletal structures and cell extensions, and nuclear DNA (cyan) with DRAQ5. Imaging was performed using high‐content confocal microscopy. Representative 700 µm stack, 4 field confocal microscopy images from a well of a 384‐well plate illustrate the distinct morphological characteristics of LN229 cell lines and patient‐derived GBM cells cultured in 3D hydrogel, Scale bar 500 µm. The LN229 cell line exhibits a homogeneous phenotype with minimal variation in filament structure. In contrast, patient‐derived cells display notable heterogeneity, consistent with the biological variability expected in our 3D hydrogel platform. Despite this variability, patient cells demonstrate robust formation of extensive filamentous networks and spheroid‐like structures, emphasizing the platform's capacity to replicate in vivo‐like tumor microenvironments. After image segmentation, quantitative assessment of GBM morphometric characteristics is conducted using two specialized surface algorithms. The exemplary image displays patient‐derived GBM cells cultured in the 3D hydrogel, stained for Phalloidin and DRAQ5. A Gaussian filter is applied to minimize over‐quantification of subcellular features, followed by appropriate thresholding and morphological operations to separate and characterize individual cellular features: one algorithm for spheroid‐like structures and another for total filament volume. In the end of the analysis pipeline, we can reliably compare different wells with different single or combinatorial treatments with different samples (e.g., patients) for their morphometric characteristics that are indicative of their pathological states. Scale bar 200 µm. All images are for representation purposes. A schematic element of this figure was created with BioRender.com (academic license).

Hydrogels consisting of starPEG‐peptide conjugates, maleimide‐functionalized heparin, cell‐adhesive RGDSP peptides, and the cells to be embedded were handled using automated liquid‐handling systems, ensuring even cell distribution and preventing cell settling. The mixture was dispensed into a low‐volume 384‐well plate at 5 µL/well. We overlaid the hydrogels with 20 µL of culture medium per well to maintain the cell viability (Figure 1). Because our hydrogel precursor mixes in < 30 s per well and polymerizes in <5 min, the entire 384‐well plate can be seeded in a single liquid‐handling run, outpacing other custom hydrogel platforms that require manual casting or multi‐step gelation. The hydrogels were cultured for 7 days, overlaid with culture medium, and examined for cellular distribution and morphology. Over the 7‐day culture period, LN229 cells predominantly displayed a filamentous morphology with occasional spheroid structures. Filamentous morphology was visualized by staining with phalloidin‐FITC, which specifically binds to F‐actin, a key cytoskeletal component involved in cell shape and invasion in 3D environments. Filamentous formations (Figures 1; S1 and S2) suggested a migratory and invasive phenotype driven by enhanced cell motility and cell‐matrix interaction. In contrast, NU01520 GBM cells exhibited both filamentous morphologies and robust spheroid formations, reflecting a more heterogeneous phenotypic profile (Figure S2). To assess these behaviors quantitatively, we measured spheroid count, individual and total spheroid volume, and total filament volume across all wells. These metrics were obtained through an integrated high‐throughput pipeline combining automated liquid handling, high‐content confocal microscopy Opera Phenix (Revvity), and batch 3D image analysis (Imaris), enabling reproducible and unbiased quantification in a single run (Figures 1; S1). For LN229, the variance between wells was 6.6%, and for patient‐derived cells, it was 12.8% (Figure S2B).

Complementing existing biomaterial GBM models, organoids, and BBB‐on‐chip systems, we deliver a plate‐native, screening‐ready starPEG–GAG 3D GBM culture platform that produces low‐volume, automated, multiplexed readouts from patient‐derived cells in ∼7 days. This chemically defined platform adds standardization and throughput while preserving key 3D phenotypes, enabling reproducible head‐to‐head therapeutic comparisons and rapid iterations.

### 2.2 3D‐HTGBM Show Resemblance to Human Primary and Recurrent GBM Biological Pathway Signatures

**FIGURE 2:**
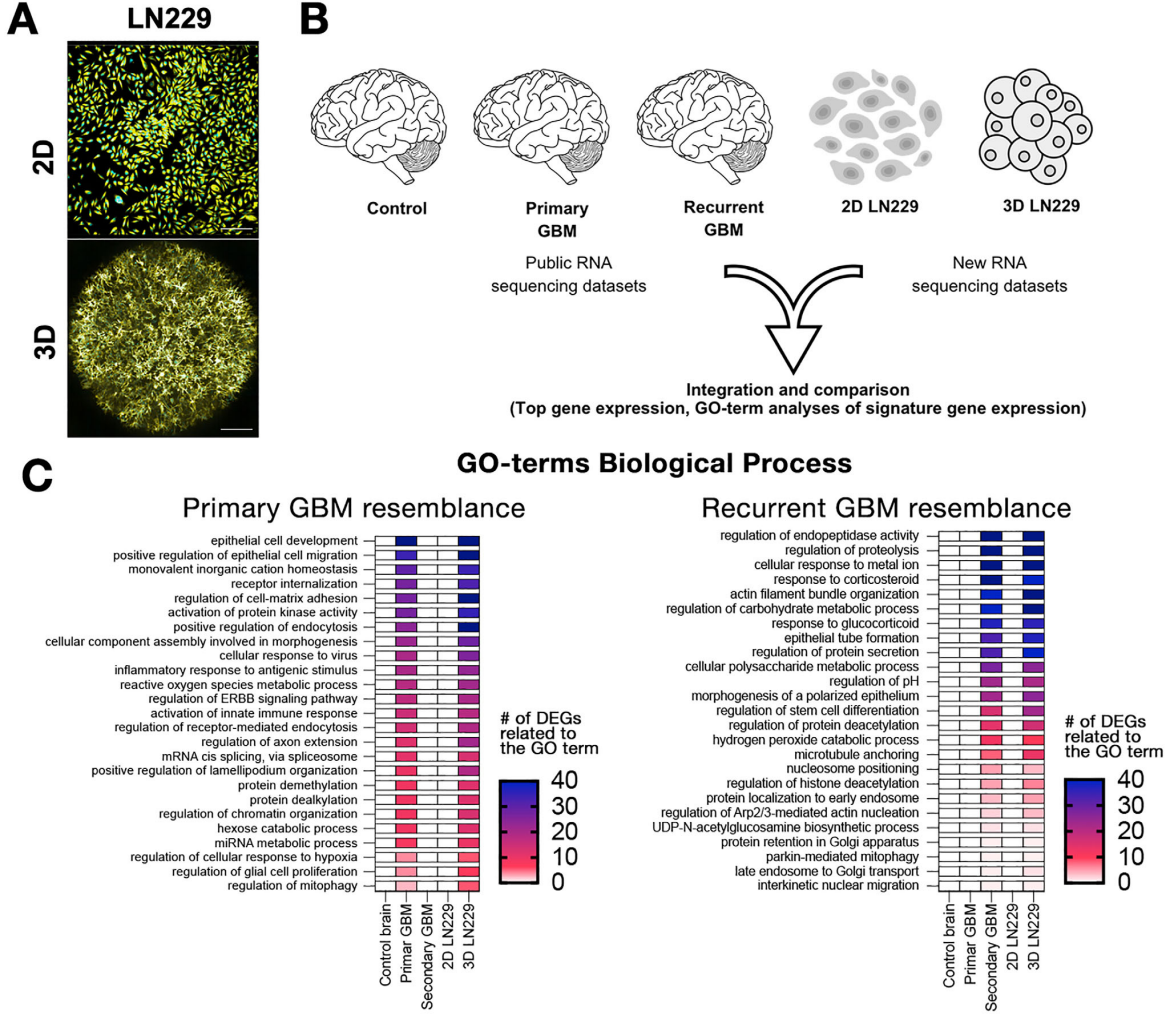
Transcriptomics analyses of 2D vs 3D GBM cell culture reveals representation of human tumor molecular programs in 3D‐HT‐GBM model. (A) Representative images of LN229 cells cultured in 2D monolayers (scale bar: 100 µm) and 3D hydrogels (scale bar: 500 µm) after 7 days. F‐actin is stained with phalloidin‐FITC (yellow), and nuclei with DRAQ5 (cyan). LN229 cells displayed extensive filamentous protrusions in 3D. In 2D, this cell type exhibited flattened morphologies. Confocal imaging revealed enhanced structural complexity in 3D compared to 2D cultures. (B) Schematic representation of the study's integrative analysis. Transcriptomic profiles of 2D and 3D LN229 cultures were compared with publicly available RNA sequencing datasets from control brain tissue, primary GBM, and recurrent GBM. This analysis integrates top gene expression data and GO‐term biological process analyses to identify similarities in molecular programs between the models and human tumors. (C) Heatmaps of GO‐term enrichment analysis comparing 3D LN229 cultures with primary and recurrent GBM datasets (GSE15824). The 3D model showed significant resemblance to primary GBM in pathways such as epithelial cell development, receptor internalization, and regulation of cell‐matrix adhesion. For recurrent GBM, enriched pathways included regulation of endopeptidase activity, actin filament bundle organization, and hypoxia‐related metabolic processes. These results emphasize the superior ability of the 3D culture model to recapitulate tumor‐related biological processes compared to 2D cultures.

To evaluate the biological relevance of our 3D hydrogel platform (3D‐HT‐GBM) compared to traditional 2D cultures, we performed bulk RNA sequencing (RNA‐seq) to analyze the transcriptomic profiles (Figure 2). A key challenge in GBM research is replicating the complex gene expression patterns observed in human tumors using *in vitro* models. While 2D monolayer cultures have been extensively used, they often fail to capture the dynamic interactions between cells and their ECM, leading to gene expression profiles that poorly represent the *in vivo* conditions. Confirming this, in 2D monolayer cultures, GBM LN229 cells exhibited flattened morphologies and reduced interaction complexity compared to 3D (Figure 2A). F‐actin was visualized using phalloidin‐FITC (yellow), which labels filamentous actin to reveal cell morphology and protrusive structures, while DRAQ5 (cyan) stains nuclear DNA to identify individual cell nuclei. In contrast, 3D culture systems provide a more physiologically relevant microenvironment by mimicking the structural and biochemical complexity of native GBM. This phenotypic shift underscores a key advantage of our biomimetic platform: its ability to induce matrix‐driven invasive traits that are suppressed on rigid, planar surfaces.

To evaluate how our starPEG–GAG hydrogel affects GBM gene expression, we compared bulk RNA‐seq profiles from LN229 cells grown in standard 2D monolayers versus our 3D hydrogel (GSE290610), alongside publicly available datasets from primary and recurrent human GBM specimens (GSE15824). In the 3D hydrogel, LN229 cultures showed enrichment (FDR < 0.05) for Gene Ontology terms associated with ECM interactions and invasive behavior—such as “regulation of cell–matrix adhesion,” “receptor internalization,” and “epithelial cell development”—pathways known to drive tumor infiltration in vivo. Similarly, pathways linked to adaptive resistance in recurrent GBM, including “regulation of endopeptidase activity” and “actin filament bundle organization,” were up‐regulated in 3D versus 2D (Figure 2B,C). In contrast, these signatures were attenuated or absent in 2D cultures, underlining the hydrogel's ability to induce transcriptional programs more reflective of key GBM pathophysiology.

While LN229 is an immortalized cell line and does not fully capture the molecular heterogeneity of patient‐derived tumors, these results demonstrate that our material design promotes gene expression patterns relevant to both primary invasion and adaptive resistance, features that are difficult to reproduce in flat, rigid substrates. Notably, the enriched Gene Ontology terms identified in 3D cultures align with transcriptomic profiles of both primary and recurrent GBM from public datasets, further supporting that our hydrogel's mechanical and biochemical cues prime cells toward more pathophysiologically relevant states. To further validate these findings, future studies will extend transcriptomic profiling to patient‐derived GBM cells, enabling deeper insights into how hydrogel cues interact with patient‐specific molecular programs.

### 2.3 Differential Effects of Chemotherapeutic Agents on 3D‐HT‐GBM Hydrogel Cultures

**FIGURE 3:**
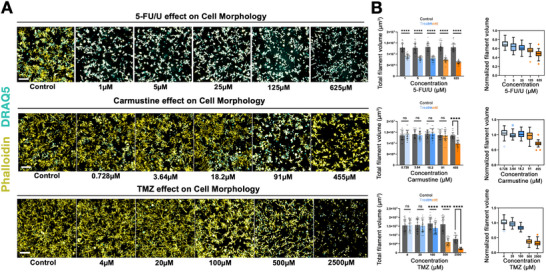
The effects of single and combinatorial chemotherapeutic agents on LN229 GBM in 3D. (A) Representative images illustrating the dose‐dependent effects of the chemotherapeutic agents 5‐FU/U, TMZ, and carmustine on invasive morphology of high‐throughput 3D hydrogel cultures of the LN229 GBM cell line. Exemplary confocal images from one field z‐stack images of 3D LN229 GBM cells are presented, showing the dose‐dependent effects of various chemotherapeutic drugs and their respective vehicle controls after a 7‐day exposure. To provide a comparison, only the lowest concentration control images are shown, as the control groups exhibited minimal variance. yellow: Phalloidin‐FITC, cyan: Draq5, scale bar 200 µm. (B) Quantification of total filament volume of LN229 cells in 3D hydrogel cultures upon treatment with chemotherapeutic agents. Dose‐dependent curves of the respective chemotherapeutic agents on the total filament volume of LN229 cells cultured in the 3D hydrogel over 7 days. The total filament volumes are reported as the mean ± SD, calculated from 36 individual measurements. Control columns are paired with each dose of the inhibitor for direct comparison. These measurements were obtained from 3 independent experiments replicates, each comprising 12 biological replicates. The normalized filament volumes are depicted using box plots following Tukey's method, displaying data distribution and outliers. Statistical significance was evaluated with *t*‐tests, accounting for variance and sample distribution differences in each dataset with ^**^ *p* < 0.01, ^***^ *p* < 0.001, ^****^ *p* < 0.0001, and ns indicating no significance.

To validate the 3D GBM hydrogel platform for quantitative biology and drug screening, we tested the dose‐dependent effects of three clinically established chemotherapeutic agents—5‐fluorouracil/Uracil (5‐FU/U), Temozolomide (TMZ), and carmustine—on LN229 GBM cells cultured within the 3D hydrogel. We quantified filamentous protrusions as a morphological representation of the cell´s invasive behavior to evaluate the efficacy of each drug within this physiologically relevant environment (Figure 3).

Chemotherapeutic agents were prepared in appropriate solvents: 5‐FU/U in cell culture media, TMZ in DMSO, and carmustine in ethanol—and doses were selected based on published IC50 values [40, 41, 42]. The selected IC50 dose, along with two higher and two lower doses, captured a range of cellular responses. Vehicle controls matched the solvent composition of each drug to account for confounding effects. Treatments began two days after cell seeding, allowing cells to establish within the hydrogel matrix [43]. Drug treatments were refreshed daily for five days to ensure stability and physiological relevance. On the eighth day, cultures were fixed and stained with phalloidin‐ FITC and DRAQ5 for high‐content confocal imaging, and Imaris software quantified total filament volume, a marker of cytoskeletal and morphological changes (Figure 3). The filamentous morphologies observed in 3D hydrogel resemble the invasive extensions of tumor cells that remodel the surrounding ECM, a feature characteristic of the mesenchymal‐like phenotype often seen in GBM.

5‐FU/U consistently reduced filament volume across all tested concentrations (1 to 625 µm) in a dose‐dependent manner. Even at low doses, 5‐FU/U demonstrated potent effects on GBM cells, consistent with its mechanism of disrupting DNA and RNA synthesis [44, 45] (Figure 3). Vehicle controls showed minimal variability, confirming the data's reliability. TMZ reduced filament volume starting at 100 µm, with higher doses (500 and 2500 µm) showing substantial effects, including extensive cytotoxicity at the highest dose. The corresponding DMSO vehicle controls did not alter filament volume, verifying drug‐specific effects (Figures 3; S3). Carmustine significantly reduced filament volume only at its highest dose (455 µm). The limited impact at lower doses was likely due to the hydrogel matrix's density reducing drug penetration, the drug's high potency threshold, or enhanced survival mechanisms within the 3D environment. Ethanol vehicle controls showed no morphological effects, confirming the results' specificity to carmustine (Figures 3; S3). To ensure statistical rigor and reproducibility of the drug treatment findings, we used three independent biological replicates of 3D cultures established on different days with the same conditions. Additionally, each experiment included 12 biological replicates to evaluate the precision and consistency of measurements.

The 3D HT GBM platform effectively captured the dose‐dependent effects of chemotherapeutic agents, demonstrating its value in systematically analyzing their cytomorphological impacts. Although total filament volume is not yet a standard readout, it has been utilized as a quantitative metric to assess invasive cellular behavior in 3D environments [46]. This approach is particularly relevant for GBM, where not just proliferation but also invasive recurrence and therapy resistance are key concerns [47].

### 2.4 Effect of Combined Chemotherapeutic Agents on LN229 Filament Volume in 3D Hydrogels

**FIGURE 4:**
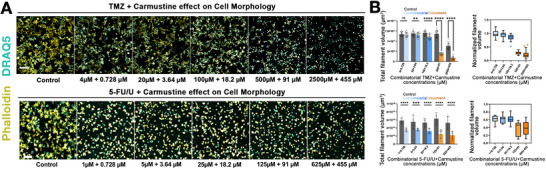
The effects of TMZ and 5‐FU/U in combination with carmustine on LN229 GBM in 3D. (A) Representative images illustrating the dose‐dependent effects of TMZ + carmustine and 5‐FU/U + carmustine combinations on high‐throughput 3D hydrogel cultures of the LN229 GBM cell line. Confocal z‐stack images from one field are presented, showing cell morphology and filamentous structures after a 7‐day exposure. TMZ and 5‐FU/U were applied in combination with escalating doses of carmustine, with the respective vehicle controls shown for comparison. DRAQ5 (cyan) stains nuclei, and Phalloidin‐FITC (yellow) highlights filamentous structures. Scale bars: 200 µm. (B) Quantification of total filament volume of LN229 cells in 3D hydrogel cultures treated with combinations of TMZ + carmustine or 5‐FU/U + carmustine. Total filament volumes were quantified for each treatment and are presented as mean ± SD. Dose‐dependent effects on filament volume are shown for each drug combination, with statistical comparisons to control groups. The TMZ + carmustine experiment data were obtained from three independent experiments, each with 12 biological replicates per condition. The 5‐FU/U + carmustine combination data were from a single experiment with 12 biological replicates per condition. Normalized filament volumes are depicted as box plots using Tukey's method to visualize data distribution and outliers. Statistical significance was assessed using *t*‐tests, with ^**^ *p* < 0.01, ^***^ *p* < 0.001, ^****^ *p* < 0.0001, and ns indicating no significance.

Since lower doses of carmustine and TMZ individually had minimal impact on the total filament volume of tumor cells in 3D hydrogels (Figure 3), we investigated whether combining these agents could better modulate invasive tumor behavior. Combining TMZ and carmustine, with distinct mechanisms of action, aims to overcome resistance to monotherapies and enhance therapeutic efficacy within the 3D tumor microenvironment. At the lowest tested concentrations (4 µm TMZ and 0.728 µm carmustine), the combination did not elicit a statistically significant change in filament volume compared to the vehicle control. Similarly, the individual drugs at these low doses exhibited negligible effects. However, the combination of 20 µm TMZ and 3.64 µm carmustine resulted in a notable reduction in filament volume relative to each single‐agent condition at the same concentrations, indicating a combination effect on invasive morphology. At higher doses, the combination of 100 µm TMZ and 18.2 µm carmustine produced a significant reduction in total filament volume, whereas in prior experiments, only 100 µm TMZ alone significantly downregulated filament volume. Carmustine at this dose alone did not elicit significant effects. A further reduction was observed at the combination of 500 µm TMZ and 91 µm carmustine. At the highest tested concentrations (2500 µm TMZ and 455 µm carmustine), the combination achieved a substantial reduction in filament volume. However, vehicle controls also exhibited toxicity at these concentrations. The experiment was conducted independently three times, each with 12 biological replicates per condition (Figure 4A,B). Overall—without inferring pharmacologic synergy‐ our data indicate that, across the mid‐dose range, the TMZ–carmustine combination yields a reproducible, greater‐than‐monotherapy reduction in invasive filament burden within this ECM‐defined 3D GBM model. Due to the constraints of the 384‐well layout, we were required to conduct monotherapy and combination conditions on separate plates, albeit on the same day. Consequently, we did not perform synergy modeling, which necessitates plate‐matched baselines to prevent intra‐plate bias. Future experiments with plate‐matched conditions will facilitate rigorous interaction analysis.

To probe combination effects on invasion, we next evaluated the combined impact of 5‐FU/U and carmustine on the total filament volume after TMZ and carmustine co‐treatment. While 5‐FU/U exhibited a dose‐dependent decrease in filament volume across all tested concentrations, carmustine only significantly reduced the total filament volume at the highest dose of 455 µm, possibly due to inherent resistance mechanisms (Figures 3; 4A,B). Notably, the combination of 5‐FU/U and carmustine resulted in a significant reduction of the total filament volume across all tested concentrations, including the lower doses where carmustine alone had previously shown no effect (Figure 3). While formal synergy modeling was not conducted, the greater‐than‐monotherapy reduction across multiple doses is consistent with a potential supra‐additive (synergy‐like) combination effect; we prudently refer to this as a “combination effect.” The combination of the drugs at the equivalent IC50 dose level (25 µm 5‐FU/U and 18.2 µm carmustine) led to a notable reduction in filament volume. Further increases in the combination dose continued to decrease the filament volume (125 µm 5‐FU/U + 91 µm carmustine), but this trend eventually plateaued, especially at the highest tested combination (625 µm 5‐FU/U + 455 µm carmustine), where no substantial additional decrease was observed. This plateauing suggests a saturation of the drug's impact, where higher concentrations do not elicit significantly greater effects on the cytoskeletal organization. The data for the 5‐FU/U and carmustine combination were obtained from a single experiment with 12 biological replicates per condition. This preliminary setup provided sufficient data points for initial insights. Future biological replicates are planned to validate these findings. These results align with our earlier observations, which showed that 5‐FU/U alone was effective across all doses, while TMZ had a more threshold‐dependent response, and carmustine was only effective at the highest dose. Together, these findings indicate a combination effect in reducing invasive morphology and illustrate that the 3D GBM platform can reliably assess combinatorial drug effects. Formal synergy analysis was not conducted because monotherapies were repeated in three experiments, whereas the combination was tested only once (12 wells per condition). However, the filament volume data alone provide a robust indication of the combination's impact on invasive behavior in our 3D GBM model.

While the combination of TMZ and 5‐FU/U was not evaluated in 3D LN229 GBM, it is a promising candidate for future investigation. As part of our ongoing efforts to expand the capabilities of this platform for personalized, multi‐drug screening, we intend to assess the efficacy of this drug pairing using patient‐derived GBM models.

### 2.5 Transcriptomics Analysis of 5‐FU/U and Carmustine Response in 3D LN229 GBM model

**FIGURE 5:**
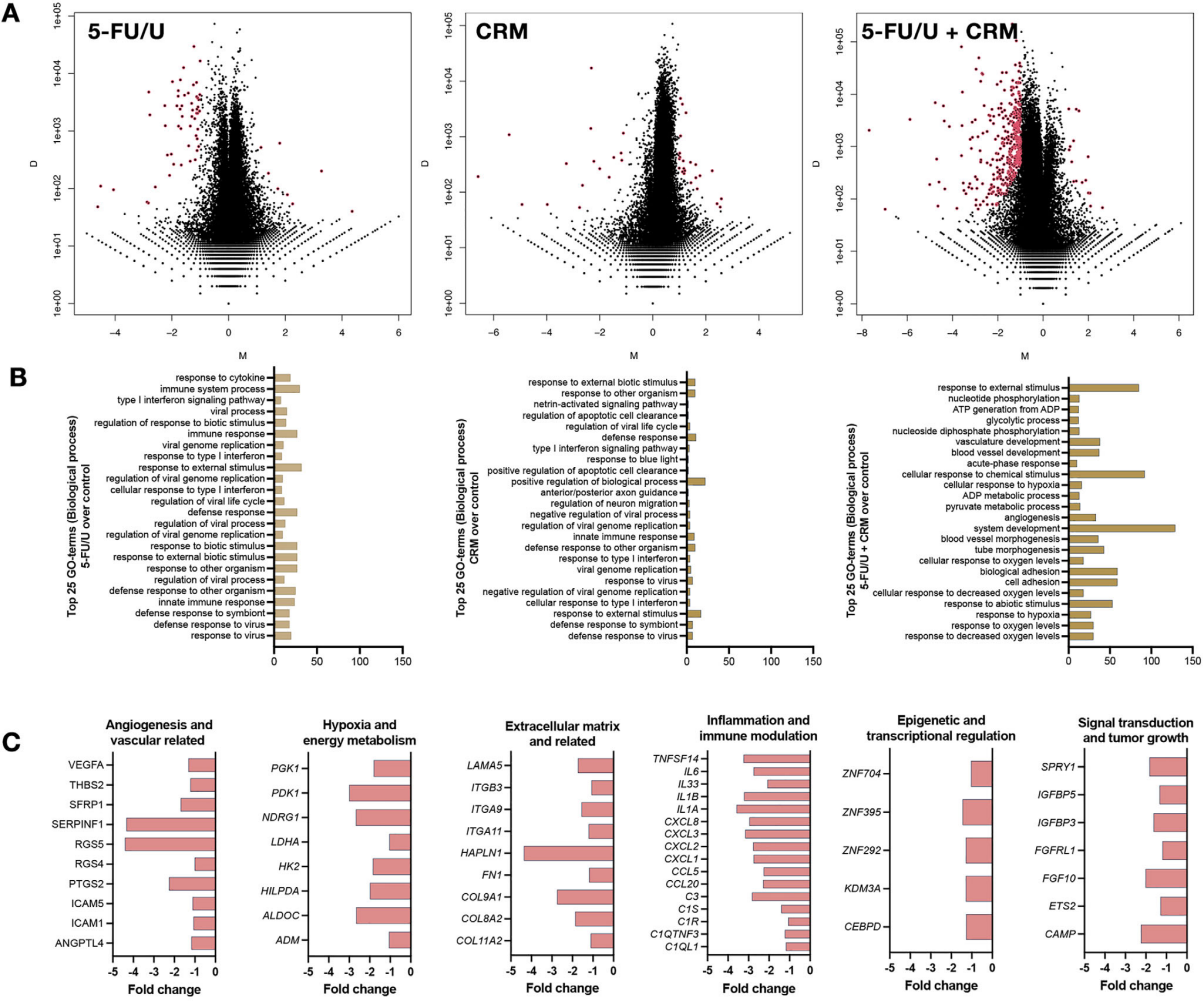
Transcriptomics analysis of 5‐FU/U and carmustine response in 3D LN229 GBM hydrogel. (A) Volcano plots illustrating differential gene expression in LN229 cells treated with 5‐FU/U (left), carmustine (CRM, middle), or a combination of 5‐FU/U + CRM (right) in 3D hydrogel cultures after 7 days. Red dots indicate significantly upregulated or downregulated genes (*p* < 0.05, fold change > 2), with the combination treatment showing the most extensive transcriptional changes. (B) Gene Ontology (GO) enrichment analysis of the top 25 biological processes associated with differentially expressed genes (DEGs) in each treatment condition. 5‐FU/U treatment predominantly affected immune‐related pathways, such as interferon signaling and cytokine response. CRM treatment modulated pathways related to cell stress responses, including apoptosis and biotic stimuli. The combination of 5‐FU/U and CRM activated additional metabolic and hypoxia‐related pathways, including ATP generation and oxygen level responses, indicating a synergistic effect. (C) Heatmaps of key DEGs grouped into six functional categories: angiogenesis and vascular‐related genes (e.g., *VEGFA*, *THBS2*), hypoxia and energy metabolism (e.g., *PGK1*, *LDHA*), extracellular matrix regulation (e.g., *FN1*, *ITGA1*), inflammation and immune modulation (e.g., *CXCL12*, *IL6*), epigenetic and transcriptional regulation (e.g., *ZNF395*, *KDM3A*), and signal transduction and tumor growth (e.g., *FGFR1*, *SPRY1*). Fold changes demonstrate the distinct and overlapping transcriptional responses across treatments. These results underscore the combination treatment's ability to modulate diverse tumor‐related pathways more effectively than individual drugs.

To investigate the molecular mechanisms underlying the observed cytomorphological changes in LN229 cells treated with 5‐FU/U and carmustine, we performed transcriptomic analysis using cells cultured in the 3D hydrogel system (Figure 5).

This analysis aimed to define distinct transcriptional signatures modulated by these drugs, either individually or in combination, providing insights beyond visible phenotypic changes. Differential gene expression analysis revealed significant alterations across all treatment conditions (Figure 5A), with the combination treatment (5‐FU/U + carmustine) inducing the most extensive transcriptional shifts. Gene Ontology (GO) enrichment analysis highlighted unique biological processes associated with each treatment (Figure 5B). Cells treated with 5‐FU/U showed an upregulation of immune‐related processes, including interferon signaling and the response to cytokines. Carmustine‐treated cells exhibited pathways involved in cellular responses to external biotic stimuli and apoptosis regulation, suggesting its role in promoting tumor cell stress responses. The combination treatment further enhanced pathways related to hypoxia, metabolic reprogramming, and ATP generation, indicating a synergistic effect in targeting tumor cell metabolism and survival mechanisms.

We further categorized the differentially expressed genes (DEGs) into key functional groups to determine the cellular processes affected by the treatments (Figure 5C). In the angiogenesis and vascular‐related category, *VEGFA* and *THBS2* were prominently downregulated, suggesting anti‐angiogenic effects. Hypoxia and energy metabolism pathways were significantly modulated, with genes such as *PGK1* and *LDHA* being upregulated in response to the combination treatment. ECM‐related genes, including *LAMA5* and *FN1*, were differentially expressed, reflecting alterations in tumor‐stroma interactions. Inflammation and immune modulation were enriched with changes in *CXCL12* and *IL6* expression. Additionally, epigenetic and transcriptional regulators, such as *ZNF395* and *KDM3A*, were altered, along with signal transduction genes like *FGFR1* and *SPRY1*, supporting multifaceted drug‐induced molecular signatures on tumor cell biology. These findings demonstrate that our treatments effectively target tumor‐related pathways, which are manifested more prominently in the 3D hydrogel model. The observed transcriptional changes are likely driven by the hydrogel's independently controlled matrix cues: stiffness by crosslink density, adhesion by RGD, degradability via the MMP‐cleavable linker on PEG, and swelling constrained by the solids window (screening condition γ = 1.50; 2.8% w/v; G′ ≈ 1 kPa). These parameters are known to influence integrin/FAK‐Rho signaling and mechano‐transduction, consistent with the 3D upregulation of ECM‐remodeling and invasion pathways observed here [23, 48, 49, 50].

A key finding was the suppression of *VEGFA*, a major pro‐angiogenic factor secreted by several cell types, including astrocytes [51, 52], which is known to modulate vascular components in the GBM microenvironment. The concurrent downregulation of other vascular‐related genes, including *ICAM1* and *THBS2*, reflects the therapy's ability to disrupt the vascular networks that sustain tumor growth and invasion. These changes are especially critical in GBM, where extensive vascularization is a hallmark of disease progression and resistance to therapy [53]. Although we did not externally impose or directly measure hypoxic conditions, our transcriptomic analysis of the combination treatment indicated robust modulation of hypoxia‐related metabolic pathways. GBM tumors rely on hypoxic adaptation to thrive in their nutrient‐deprived microenvironments, making the alteration of genes such as *PDK1*, *NDRG1*, and *ALDOC* central to their survival [54, 55, 56]. In our dataset, their downregulation suggests that the therapy not only inhibits angiogenesis but also interferes with metabolic reprogramming, potentially rendering tumor cells more vulnerable to therapeutic stress. We therefore interpret these changes as activation and therapeutic disruption of cell‐intrinsic hypoxia signaling elicited by the 3D ECM context and treatment‐induced stress under nominally normoxic culture—an effect frequently observed in physiologically relevant GBM models [21]. Accordingly, these signatures should be viewed as markers of metabolic and microenvironmental adaptation rather than evidence of experimentally induced oxygen depletion. It is also important to note that hypoxia‐associated pathways exert pleiotropic effects beyond oxygen sensing—intersecting with key regulators of cell survival, invasion, and therapy resistance [57, 58]. Crosstalk between HIF signaling, PI3K–AKT–mTOR, NF‐κB, and MAPK pathways can modulate energy metabolism, redox balance, and stemness, thereby amplifying adaptive responses even under nominally normoxic conditions. These interactions may therefore contribute to the multidimensional transcriptional changes observed in our 3D cultures. Future work incorporating oxygen‐controlled culture and/or real‐time hypoxia sensors will be important to directly validate and extend these observations.

Similarly, the suppression of ECM‐related genes like *FN1*, *ITGA9*, and *LAMA5* highlights a disruption in the tumor's invasive machinery, which depends on ECM remodeling for infiltration into surrounding tissues [59, 60]. By targeting these processes, the treatment demonstrates its ability to impair not only tumor growth but also its invasive potential. Furthermore, the hydrogel matrix allows to control biomimetically growth factor and cytokine affinity; these soluble signaling cues are well‐known to control tumor growth [31, 61]. Indeed, we also observed downregulation of inflammatory mediators such as *IL6*, *CXCL8*, and *CCL20*, indicating a reduction in tumor‐promoting inflammation. This is significant, as inflammatory cytokines and chemokines contribute to immune evasion and support a pro‐tumorigenic microenvironment in GBM [62, 63]. The modulation of epigenetic regulators and signal transduction pathways, such as *ZNF395*, *FGFRL1*, and *KDM3A*, further reflects the combinatorial treatment's impact on transcriptional networks that drive tumor plasticity and progression.

Transcriptomic profiling in this study was limited to a single cell line and only the effects of three drugs; however, ongoing follow‐up investigations aim to expand the analysis to a broader panel of patient‐derived GBM cells and multiple chemotherapeutic drugs, either alone or in combination, to validate the molecular mechanisms underlying the observed morphological responses. Additionally, single‐cell RNA‐seq can be applied to evaluate the degree to which our model captures the intratumoral heterogeneity and diverse GBM transcriptional states observed in patients.

### 2.6 Chemotherapeutic Intervention of Patient‐Derived Primary 3D GBM Model

**FIGURE 6:**
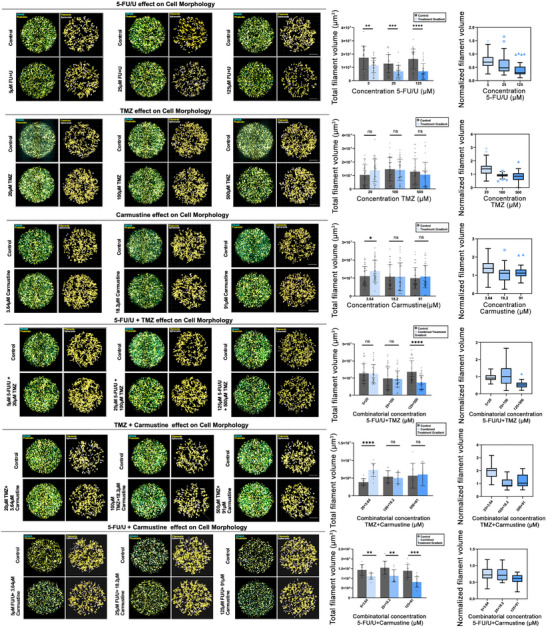
Quantitative analysis and morphological characterization of filamentous structures in patient‐derived NU01520 3D GBM cultures under chemotherapeutics. The graphs on the right panels illustrate the quantitative impacts of 5‐FU/U, TMZ, and carmustine alone as well as 5‐FU/U + TMZ, TMZ + carmustine, and 5‐FU/U + carmustine on patient‐derived 3D GBM cell cultures. The representative confocal microscopy images, paired with Imaris‐generated visualizations of filaments and spheroids on the left panel, illustrate the structural and organizational changes within the 3D cultures after 7 days of treatment. Each dose‐dependent drug treatment is paired with their respective control. Yellow: Phalloidin‐FITC, cyan: DRAQ5. Scale bar 200 µm. Quantification (right panels) of the total filament volumes are presented as the mean ± SD, with control columns paired with each dose of the inhibitor for direct comparison. The 5‐FU/U condition was tested across 4 independent experiments, and TMZ had 5 experiments, with varying numbers of biological replicates to account for Janus HTS system validation. Carmustine was evaluated using 3 independent experiments, each with 12 biological replicates. The combination of 5‐FU/U and TMZ had 3 experiments, with 12 biological replicates per condition. For the combination treatments of TMZ and 5‐FU/U with carmustine, 12 biological replicates per condition were used. The normalized filament volumes are depicted using box plots following Tukey's method, which displays the data distribution and outliers. Statistical significance was evaluated using *t*‐tests, accounting for variance and sample distribution, ^*^ *p* < 0.05, ^**^ *p* < 0.01, ^***^ *p* < 0.001.

To extend our findings beyond the LN229 cell line, we next evaluated the effects of single or combinatorial chemotherapeutic agents in 3D hydrogel cultures of patient‐derived GBM cells. Patient‐derived NU01520 cells (see methods for patient sample description) cultured in the 3D hydrogel system exhibited substantial morphological heterogeneity, including diverse filamentous structures and spheroid morphologies, reflecting the inherent diversity of tumor cell populations. Chemotherapeutic agents were administered at concentrations near their published IC50 values, and the dosing strategy was refined for clinical relevance. Treatment with 5‐FU/U resulted in a significant, dose‐dependent reduction in total filament volume at all tested concentrations (5, 25, and 125 µm), consistent with results from LN229 cells, highlighting 5‐FU/U's potent cytotoxic effects in disrupting cytoskeletal structures (Figures 6; S4). In contrast, TMZ treatment, at concentrations of 20, 100, and 500 µm, did not significantly impact total filament volume in NU01520 cells, underscoring the therapeutic resistance of this patient‐derived cell line compared to the more homogeneous LN229 model (Figure 6). To validate the robustness of the automated cell culture system (Janus HTS), we performed multiple independent experiments with biological replicates of 5‐FU/U (*n* = 4) and TMZ (*n* = 5). Confirming our previous results, carmustine at concentrations of 3.6, 18.2, and 91 µm also failed to produce significant changes in filament volume. Interestingly, a slight increase in filament volume at the lowest carmustine concentration suggested potential compensatory survival mechanisms akin to the variability often observed in patient‐derived cell models (Figure 6). We evaluated the effects of carmustine effects using 3 independent experiments, each with 12 biological replicates.

To capture the full morphological diversity of patient‐derived GBM cultures, we measured not only total filament volume but also quantitative parameters of spheroid formation, including spheroid count, mean, and total spheroid volume. These additional metrics allowed us to characterize structural changes that are more prominent in primary GBM cells compared to established cell lines.

**FIGURE 7:**
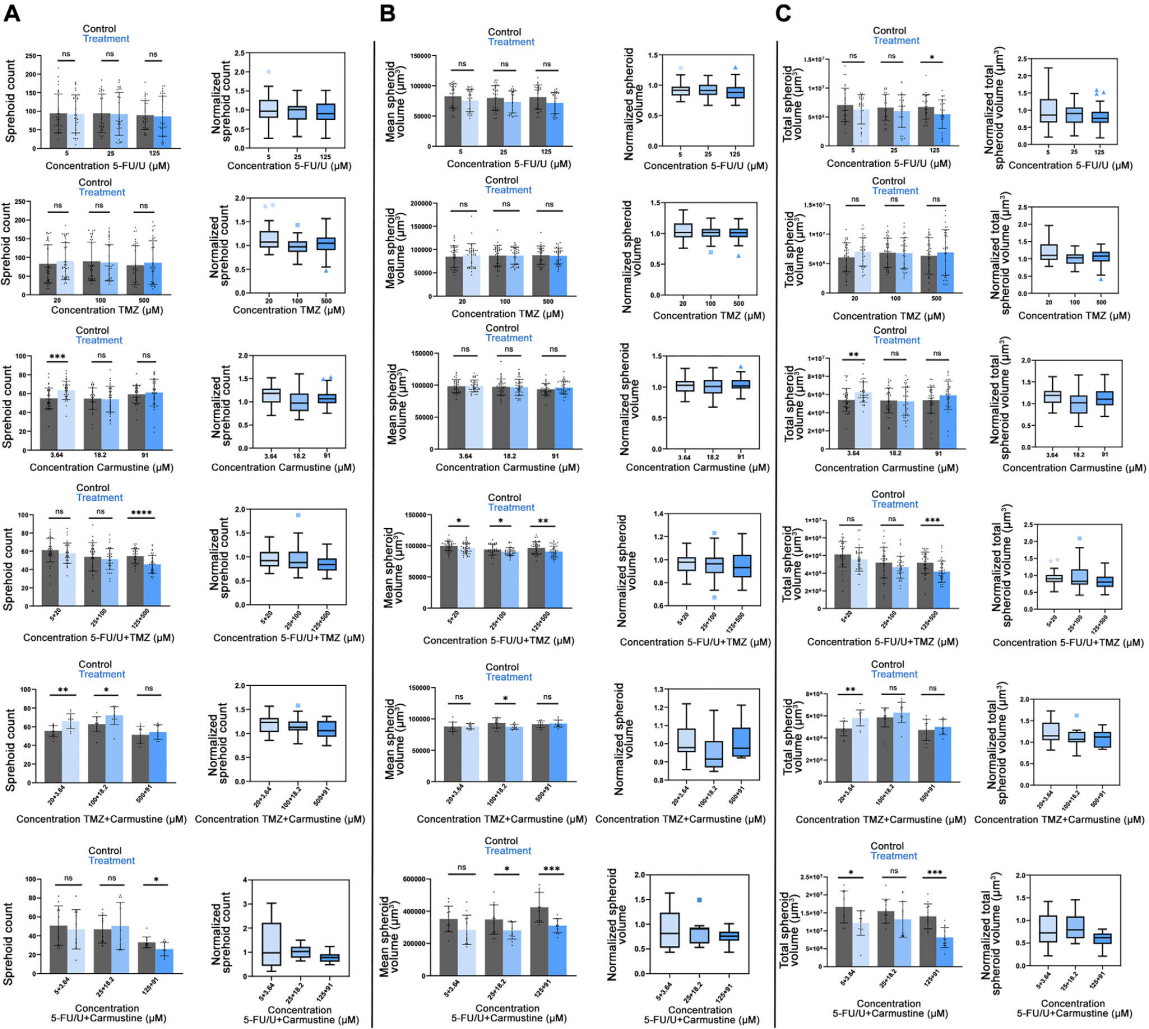
Impact of individual and combination chemotherapeutic agents on spheroid formation in patient‐derived 3D GBM cultures. This figure depicts the quantification graphs for the changes in spheroid formation ((A) Spheroid count; (B) Mean spheroid volume, (C) Total spheroid volume) observed in patient‐derived 3D GBM cultures following treatment with individual chemotherapeutic agents (5‐FU/U, TMZ, and carmustine) and their combinations (5‐FU/U and TMZ, TMZ and carmustine, and 5 FU/U + carmustine). The data are presented as the mean ± SD, with control columns paired alongside each drug concentration for direct comparison. The 5‐FU/U condition was tested across 4 independent experiments, and TMZ in 5, whereas carmustine in *n* = 3 experiments. The combination of 5‐FU/U and TMZ had 3 independent experiments, with 12 biological replicates per condition. For the combination treatments of TMZ and carmustine and 5‐FU/U + carmustine, 12 biological replicates per condition were used. Data normality was assessed to determine the appropriate statistical test—Welch's *t*‐test for normally distributed data and the Mann–Whitney *U* test for non‐parametric data. Significance thresholds were set at *p* < 0.05, *p* < 0.01, *p* < 0.001, and *p* < 0.0001. Normalized spheroids count data are presented as box plots employing Tukey's method.

While 5‐FU/U treatment significantly reduced filament volume, spheroid metrics (count, mean volume, and total volume) showed minimal changes. Only the highest concentration of 125 µM reduced total spheroid volume, indicating that 5‐FU/U primarily disrupts filamentous structures rather than spheroid formation or proliferation (Figures 7; S4). Consistent with the filament volume data, TMZ and carmustine also failed to reduce spheroid count, mean spheroid volume, or total spheroid volume in patient‐derived 3D cultures (Figure 7). However, when combined, 5‐FU/U and TMZ reduced filament volume, spheroid count, and total spheroid volume only at the highest dose (125 µm 5‐FU/U and 500 µm TMZ), whereas mean spheroid volume was consistently reduced across all concentrations. The differential sensitivity observed across morphological parameters highlights the versatility of our 3D hydrogel platform in detecting dose‐dependent and feature‐specific drug responses, which reflects the complex behavior of GBM cells within a physiologically relevant environment (Figures 6 and 7; S4). We performed the 5‐FU/U and TMZ combination treatment in 3 independent experiments, with 12 biological replicates per condition.

The TMZ and carmustine combination did not significantly impact filament volume and any spheroid metrics across tested concentrations. Notably, carmustine at lower concentrations increased total filament volume, spheroid count, and total volume, potentially indicating compensatory proliferative mechanisms or cellular redistribution into smaller spheroids in response to cytotoxic stress (Figures 6, 7). In contrast, the 5‐FU and carmustine combination exhibited measurable suppression across most parameters. Filament volume was consistently reduced across all doses, spheroid count decreased only at higher concentrations, mean spheroid volume dropped at the higher doses, and total spheroid volume was suppressed at both low and high concentrations. These coordinated changes indicate a broader suppression of invasive architecture and multicellular growth than either agent alone (Figures 6 and 7). Consistent with these phenotypes, transcriptomic profiling under matched conditions revealed a coordinated downregulation of invasion‐ and adhesion‐associated ECM genes, including *FN1*, *ITGA9*, and *LAMA5* (Figure 5C). These changes indicate impaired cell–matrix interactions, which are essential for tumor cell adhesion, migration, and the formation of invasive protrusions in 3D environments. The suppression of these ECM components provides a molecular basis for the phenotypic observations, suggesting that the combination treatment disrupts the structural and signaling cues required for efficient infiltration and multicellular expansion within the hydrogel matrix. Although this phenotypic pattern aligns with complementary drug effects, we avoid attributing specific molecular mechanisms based solely on imaging. To enhance our understanding, future research will include pathway‐level assays within the same 3D matrix.

Conversely, TMZ and carmustine had limited effects on filament volume in patient‐derived NU01520 cells, reflecting their inherent resistance to standard therapies. These findings highlight the ability of the 3D hydrogel system to detect the differential efficacy of drugs based on cell type, offering a robust platform for translational research. Notably, combining 5‐FU/U with carmustine produced consistent, multi‐parameter reductions in invasive architecture across both LN229 and patient‐derived GBM cultures. These data indicate a robust additive benefit of the combination in a 3D ECM‐defined context and prioritize it for follow‐up with formal interaction modeling and pathway assays, rather than constituting a definitive claim of pharmacologic synergy.

Also, high‐dose combinations of 5‐FU/U and TMZ led to significant reductions in filament volume, spheroid count, and spheroid volume, indicating that TMZ may sensitize GBM cells to 5‐FU/U. In contrast, the combination of TMZ and carmustine showed no significant improvement over monotherapies, further emphasizing the challenges posed by GBM's adaptive resistance mechanisms. These results illustrate the need for careful optimization of drug combinations and dosing regimens within physiologically relevant models. By measuring multiple morphological changes, not just cell death, we provide a more comprehensive way to evaluate and optimize combination therapies for GBM.

### 2.7 Patient‐Specific Morphological Diversity in 3D Hydrogel Cultures Reflects the *In Vivo* Adaptability of GBM

**FIGURE 8:**
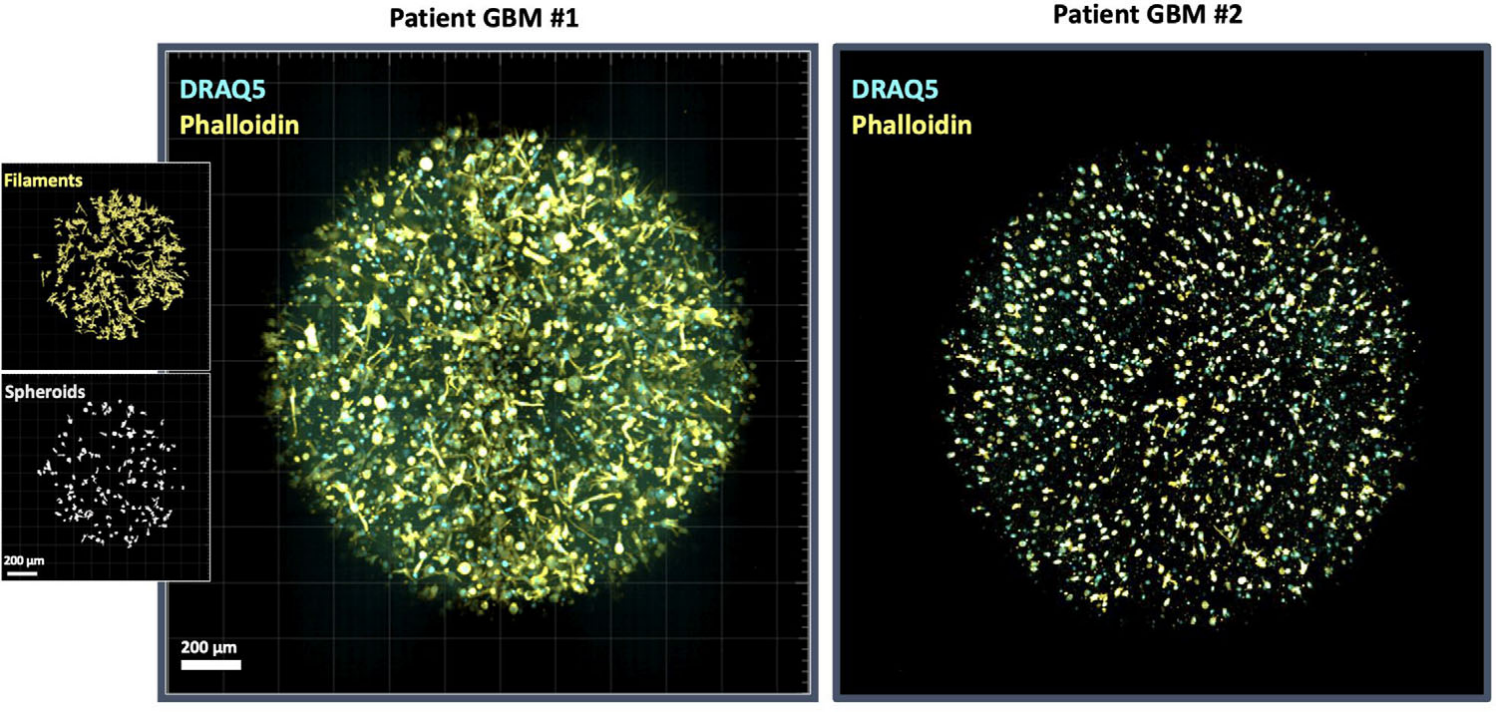
Morphological diversity of GBM cells from different patients in 3D hydrogel cultures. NU01520 patient‐derived GBM cells (Patient GBM #1) formed an extensive filamentous network with numerous spheroids after 7 days in 3D hydrogel culture, as determined by the Imaris image segmentation pipeline. KUGBM8 patient‐derived GBM cells (Patient GBM #2) exhibited a lesser extent of network formation, with a predominance of spheroid structures after 7 days in the same 3D hydrogel culture conditions. Yellow: Phalloidin‐FITC, cyan: Draq5, scale bar 200 µm.

To explore the impact of inter‐patient heterogeneity, we cultured a second patient‐derived GBM cell line, KUGBM8 (histological grade IV primary GBM from the temporooccipital region of a 40‐year‐old male with no prior treatments administered, carrying wild‐type IDH1, methylated MGMT promoter, EGFR amplification (score 3), negative for PTEN (score 32), and positive for TP53 mutation (score 3)), in the 3D hydrogel system under identical conditions. These cells exhibited markedly reduced filamentous network formation compared to NU01520 cells (see methods for patient details), despite forming a substantial number of spheroids (Figure 8). The phenotypic variability observed between these two patient‐derived cell lines reflects the adaptability of GBM cells to shifting microenvironmental cues, mirroring the complexity of in vivo tumor settings. Such diversity underscores the need for patient‐derived models to accurately replicate tumor heterogeneity and inform therapeutic strategies.

While detailed data are presented here for two patient‐derived GBM lines, all additional isolates tested to date similarly formed spheroids or invasive filaments within the hydrogel matrix, with low well‐to‐well variability. This consistency across genetically distinct samples underscores the reproducibility and robustness of the platform.

### 2.8 Limitations and Future Directions

Our results emphasize the importance of refining 3D microenvironments to better emulate human conditions, ultimately driving advancements in GBM treatment. The presented platform recapitulates tumor‐associated gene programs and morphologies that are correlated with in vivo GBM phenotypes, providing a scalable scaffold for hypothesis testing prior to in vivo validation. While *in vivo* models are considered the gold standard for therapeutic prediction, our platform demonstrates the ability to capture key features of drug response, including the suppression of invasive morphology, by utilizing patient‐derived GBM cells in a defined microenvironment. We also recognize that the single‐component hydrogel does not yet incorporate key non‐tumor elements such as vascular or immune cells. However, the modular chemistry of starPEG–GAG hydrogels enables straightforward addition of GAG‐bound growth factors to mimic paracrine signaling, incorporation of vascular‐adhesion peptides for microvascular network formation, and integration with microfluidic perfusion to emulate interstitial flow. These biomaterial extensions may allow stepwise reconstruction of GBM's complex microenvironment in a high‐throughput, fully automated format, which could be complemented by a systematic comparison with ECM‐free spheroids.

## 3 Conclusion

Our 3D hydrogel culture system provides a robust, scalable, and physiologically relevant platform for preclinical GBM drug testing. The platform integrates automated imaging, high‐content image analysis, and transcriptomic profiling within a matrix‐metalloproteinase–responsive starPEG–GAG hydrogel. This combination enables quantitative assessment of invasive morphologies, such as filamentous protrusions and spheroid formation, together with the evaluation of drug‐induced transcriptional responses. We show that filament and spheroid metrics capture dose‐dependent effects of monotherapies and reveal pronounced combination effects for 5‐FU/U plus carmustine—insights that are difficult to obtain from conventional viability assays or ECM‐free spheroids. Importantly, the hydrogel microenvironment alone was sufficient to induce transcriptional programs in LN229 cells that align with those observed in primary and recurrent GBM, demonstrating that matrix mechanics and ligand presentation can prime cells toward more pathophysiologically relevant states. This is particularly valuable in settings where speed and throughput are essential, and where standard 2D or ECM‐free models fall short in recapitulating GBM heterogeneity and adaptive resistance. Our platform bridges the gap between simplistic *in vitro* assays and low‐throughput *in vivo* models by enabling reproducible, high‐content evaluation of tumor‐associated behaviors and gene programs. It provides a versatile scaffold for hypothesis testing prior to *in vivo* validation and lays the foundation for future expansions, including co‐culture with other cell types, perfused tissue‐on‐chip models, and single‐cell resolution profiling. Our model exemplifies the power of engineered microenvironments in capturing complex GBM biology and supporting the development of predictive pre‐clinical translation systems as well as patient‐relevant companion diagnostic tools.

## 4 Materials and Methods

### 4.1 Ethical Statement

All research involving human‐derived samples was conducted in compliance with ethical guidelines and approved by institutional review boards (IRBs) at the respective institutions. The patient‐derived GBM cells (NU01520) were collected under the IRB‐approved protocol at Northwestern University, Chicago, IL, USA. Written informed consent was obtained from all patients prior to sample collection, in accordance with the Declaration of Helsinki. Tumor tissues were processed under sterile conditions to establish primary cell cultures, ensuring the preservation of cellular integrity and the anonymity of the donors. The KUGBM8 patient‐derived GBM cell line was collected and processed under IRB oversight at Koç University, Turkey, in strict adherence to both local and international ethical standards for biomedical research. The use of these samples was restricted to experimental protocols aligning with IRB‐approved guidelines, and no identifiers were linked to the donors. To confirm the absence of pathogens, all patient‐derived and established GBM cell lines underwent comprehensive pathogenicity testing. Mycoplasma contamination was routinely screened using PCR‐based methods. Viral contamination was assessed using RT‐PCR for common retroviral and adenoviral contaminants, ensuring that all cultures were pathogen‐free. These measures minimized biosafety risks and ensured the validity of the experimental results. All procedures involving LN229 GBM cells followed standard institutional guidelines for cell line research, including regular pathogenicity testing and the implementation of robust biosafety protocols approved in IPF, Dresden.

### 4.2 Preparation of Patient‐Derived Cells From Biopsies

The patient‐derived GBM cells, NU01520, used in this study originated from a 62‐year‐old male patient of White, non‐Hispanic ethnicity, who was diagnosed with grade IV GBM. The tumor exhibited significant genetic and molecular characteristics, including wild‐type IDH1/2 status, retained ATRX expression, and mutations in TP53 and PTEN. These comprehensive genetic and clinical features were analyzed to ensure a robust correlation between the in vitro modeling and the in vivo tumor behavior, thereby enhancing the translational relevance of the 3D culture experiments. Tumor tissue was collected from the operating rooms at Northwestern Memorial Hospital in Chicago, IL. All patients signed a written informed consent under the Northwestern University approved IRB protocol, STU00095863. Tumor tissue was transported from the operating room to the Nervous System Tumor Bank laboratory, where it was processed for cell culture establishment. Tissue was first manually minced using sterile disposable scalpels in a 10 cm dish. The minced tissue was then transferred to a sterile 50 mL conical containing 4 mL RPMI 1640 (Gibco; 22400089) and 100 uL collagenase (1 mg/1 mL; Sigma, C1889) for enzymatic digestion. The mixture was incubated for 1 h at 37°C. An additional 30 mL RPMI was then added to the mixture and centrifuged at 1500 rpm for 8 min at 4°C. The supernatant was aspirated and the cell pellet was resuspended with complete media (RPMI 1640, 10% fetal bovine serum (FBS, [Gibco; 16000044]), 1% sodium pyruvate (Gibco; 11360039), 1% penicillin‐streptomycin (Pen/Strep, [Gibco; 15140122]), 1% MEM Non‐Essential Amino Acids (NEAAs, [Gibco; 11140035]). Cells were then plated in Corning TC‐Treated Culture Dishes.

### 4.3 2D Culture Conditions for LN‐229 GBM Cell Line and Patient‐Derived GBM Cells

The LN‐229 GBM cell line, acquired from the American Type Culture Collection (ATCC, Manassas, VA, USA, RRID: CVCL_0393), and two patient‐derived GBM cell lines, NU01520 from Northwestern University, USA, and KUGBM8 from Koç University, Turkey, were all cultured under standardized 2D conditions to ensure reproducibility and consistency across experiments. LN‐229 cells were maintained according to the manufacturer's specifications in Dulbecco's Modified Eagle Medium (DMEM, [Sigma–Aldrich; D5796]) supplemented with 5% FBS, 1% Pen/Strep, and 1% GlutaMAX (Gibco; 35050038). Cells used for experiments were at passages 6 to 8. The patient‐derived GBM cells from NU01520 were expanded in T‐75 tissue culture flasks coated with Geltrex (Gibco; A1413301) for 1 h in an incubator (1:50 dilution in PBS [Sigma; D5652‐10x]) and cultured in RPMI 1640 Medium with HEPES, supplemented with 10% FBS, 1× NEAAs, 1 mm sodium pyruvate, and 1% Pen/Strep. Cells between passages 4 and 6 were utilized for all experiments. Similarly, the KUGBM8 patient‐derived GBM cell line from Koç University was expanded in Geltrex‐coated T‐75 tissue culture flasks and cultured in a specialized medium consisting of a 1:1 mixture of DMEM/F12 and RPMI 1640, supplemented with 1% B27 Plus (Gibco; A3582801), 0.5% N‐2 supplement (Gibco; 17502048), 5% FBS, 20 ng/mL epidermal growth factor (EGF, [PeproTech; AF‐100‐15]), 20 ng/mL basic fibroblast growth factor (FGF2, [PeproTech; 100–18B]), 1× NEAAs, 1 mm sodium pyruvate, and 1% Pen/Strep. This formulation was specifically optimized to support the growth and maintenance of primary GBM cells. Cells between passages 2 and 4 were used for experiments. LN‐229 and patient‐derived GBM cells were maintained at 37°C in a humidified atmosphere with 5% CO_2_. The culture medium was refreshed every 48 h, and cells were passaged upon reaching 70–80% confluence using StemPro Accutase (Gibco; A1110501).

### 4.4 Preparation of starPEG‐Heparin Hydrogel and Encapsulation of LN229 and Patient‐Derived GBM Cells

The gel preparation was performed in automated liquid handling systems in a GMP‐certified clean room in the Leibniz Institute for Polymerforschung, Dresden, hosting Neuron D GmbH facilities (https://neurond.de/our‐facilities). On the day of the experiment, two hydrogel precursor solutions were freshly prepared following established protocols. The first solution consisted of a matrix metalloproteinase (MMP)‐responsive star‐shaped PEG‐peptide conjugate (starPEG; multi‐arm PEG, 10 kDa per arm; JenKem Technology), as described before [64]. The second solution consisted of maleimide‐functionalized sulfated glycosaminoglycan (sGAG) heparin (HM6, 6 maleimides per ∼15 kDa chain), as per the methods of Limasale et al. [65], combined with a fibronectin‐derived RGDSP cell‐adhesive peptide (Final concentration: 1.5 mM, synthesized in‐house [64], covalently linked to the sGAG via Michael‐type addition, and a concentrated cell suspension. Cell densities for encapsulation were as follows: LN‐229 at 3000 cells per 5 µL gel, NU01520 at 8000 cells per 5 µL gel, and KUGBM8 at 8000 cells per 5 µL gel. Cells were routinely detached from culture flasks using Accutase for 5 to 8 min at 37°C in a humidified incubator with 5% CO_2_. After detachment, cells were counted using the countess automated cell counter (Thermo Fisher Scientific, DE) with Trypan Blue exclusion (1:2 dilution in PBS) to assess viability. The cell suspensions were then centrifuged at 500 g for 7 min at room temperature. Following centrifugation, concentrated cell suspensions were prepared in PBS and added to hydrogel precursor solution 2 for encapsulation. Both hydrogel precursor solutions were prepared using PBS, pH 7.4, and the gelation time was precisely controlled to 60–80 s. This was achieved by acidifying the pH of the starPEG‐peptide conjugate solution using hydrochloric acid, following the published protocols [39, 65]. For automated handling, either the Tecan Fluent 480 liquid handling system (Tecan Group, CH) or the JANUS G3 liquid handling system (Revvity, Inc., US) was utilized to sequentially transfer the stock solutions into V‐shaped wells of a 96‐well plate for thorough mixing. The resulting mixtures were subsequently transferred into a 384‐well low‐volume plate, with 5 µL allocated per well. The pre‐adjusted gelation time ensured rapid hydrogel polymerization after transfer, preventing cell sedimentation and promoting uniform cell distribution within the hydrogels. Once polymerization was complete, 20 µL of culture medium was gently layered over the hydrogel in each well. For the 3D culture of LN‐229, the cells were maintained in DMEM/F12 medium supplemented with 20% FBS, 1.5% GlutaMAX, and 1% Pen/Strep. In contrast, the 3D culture medium for NU01520 and KUGBM8 consisted of a 1:1 mixture of DMEM/F12 and RPMI 1640, supplemented with 2.5% B27, 1.25% N2, 1.5% GlutaMAX, 1.5X NEAA, 1.5 mM sodium pyruvate, 100 ng/mL FGF2, 100 ng/mL EGF, and 1% Pen/Strep. These media formulations were optimized to support the growth of the respective cells in 3D cultures. To prevent medium evaporation from the outermost wells, the peripheral rows and columns were filled with PBS supplemented with 1% Pen/Strep and excluded from hydrogel preparation. This setup allowed for the use of 240 wells per 384‐well plate for hydrogel‐based 3D cell cultures, maximizing experimental capacity while maintaining consistent culture conditions.

### 4.5 Preparation of Chemotherapeutic Agents for 3D Encapsulated Cell Treatment

For the preparation of the chemotherapeutic agents, a stock solution of 6.094 mm 5‐fluorouracil (FU, [Thermo Fisher Scientific; 227601000]) and 14.333 mm Uracil (U, [Thermo Fisher Scientific; 140770010]) was prepared in DMEM media. The two drugs were then combined and further diluted in DMEM to produce final working concentrations of 1, 5, 25, 125, and 625 µm for the combined FU/U treatment. Temozolomide (TMZ, [Sigma‐Aldrich; T2577]) was initially dissolved in DMSO (Dimethyl sulfoxide, [Sigma; D2650]) to create a 92.7 mm stock solution. This stock was then further diluted in DMEM to prepare working concentrations of 4, 20, 100, 500, and 2500 µm for use in the experiments. Carmustine (BCNU, [Sigma–Aldrich; C0400]) was dissolved in ethanol (EtOH, [VWR Avantor; 20821.365p]) at a concentration of 95.3 mm. The stock was then diluted to the final concentrations (0.728, 3.64, 18.2, 91 , and 455 µm). Following the preparation of the working concentrations, FU/U and TMZ as well as FU/U and carmustine, were combined for the experimental treatments by mixing their respective concentrations in DMEM media. Additionally, TMZ was also combined with carmustine at their corresponding working concentrations. The stock solutions were aliquoted into sterile, light‐protected tubes and stored at −20°C. To avoid freeze‐thaw cycles, single‐use aliquots were prepared and thawed only once for experimental use. Working concentrations for each drug were freshly prepared on the day of the experiment by diluting the stock solutions to the desired concentrations in DMEM media. Control wells were treated with the corresponding solvents: DMSO alone for TMZ treatments, ethanol for carmustine, and DMEM alone for FU/U. Solvent controls were matched to the final concentrations present in the drug‐treated wells to ensure accurate comparisons between drug‐treated and control conditions. For LN229 cells, following an initial 2‐ day culture period, drug treatments were introduced daily in the 384‐well plates for a duration of 5 days. In contrast, for the NU01520 and KUGBM8, after an initial 2‐day culture period, the media containing drugs were refreshed every second day for a total of 5 days. Both the LN229 cell line and patient cells remained in culture for 7 days and were fixed on the eighth day. This differential dosing schedule was maintained throughout the experiment to account for the varying growth and treatment response profiles of the different cell types.

### 4.6 Immunofluorescence Staining, Imaging, and Quantification

Following drug treatments, the 3D encapsulated cells in 384‐well plates were subjected to three washes with 20 µL of PBS containing 1% Pen/Strep. Cells were then fixed with 20 µL of 4% paraformaldehyde (PFA, [Roth; 0335.3]) for 30 min at room temperature. Post‐fixation, the cells underwent three additional washes with PBS containing 1% Pen/Strep, leaving the final wash in the wells. All steps of the fixation process were automated using the Tecan Fluent 480 liquid handling system, ensuring precision and consistency across the wells. For immunostaining, 20 µL of a staining solution was added to each well, consisting of DRAQ5 [Thermo Fisher Scientific; 62251] at a 1:500 dilution, for nuclear staining (excitation/emission: 646/681 nm), and Phalloidin conjugated to Alexa Fluor 488 [ATTO‐TEC GmbH; AD 488‐82] at a 1:200 dilution in PBS, for actin filament staining (excitation/emission: 495/519 nm). The plate was sealed and incubated at 4°C for 24 h, protected from light to prevent photobleaching of the fluorophores. Following staining, the cells were washed three times with PBS containing 1% Pen/Strep. To stabilize the samples for imaging, 20 µL of an antibiotic‐antimycotic solution [Sigma; A5955] in PBS with 1% Pen/Strep was carefully added to cover the gels. Imaging was performed immediately, or the plate was sealed with adhesive aluminum foil and stored at 4°C until imaging was conducted.

Stained hydrogels were imaged on a spinning disk confocal microscope Opera Phenix Plus (Revvity, Inc., US, RRID:SCR_021100), equipped with 10x air objectives (NA 0.3, acquisition performed with 2x binning). To minimize boundary artifacts in the 384‐well format, z‐stacks were acquired from 70 µm above the well bottom to 320 or 700 µm into the gel at a constant step size, thereby excluding the plastic interface. Image fields were centered within each well (i.e., not contiguous with the side wall) or acquired as 2×2 mosaics stitched prior to 3D image analysis. Further quantification was performed over the entire acquired volume to avoid field‐selection bias; metrics were not stratified by radial position or well location. Media volume and plate humidity were kept constant across experiments to limit evaporation‐driven edge effects.

The confocal microscopy images in this manuscript have been processed with consistent adjustments to ensure uniformity and clarity. Images obtained using the Opera Phenix system were processed with identical gamma settings and consistent minimum‐maximum thresholds for Alexa Fluor 488 and DRAQ5. As an example, for LN229 3D GBM image processing, Alexa Fluor 488 was displayed in yellow with intensity values ranging from 302 to 5283, while DRAQ5 was displayed in cyan with intensity values ranging from 285 to 542. It is important to note that the nuclear and filamentous staining appears oversaturated in some images, particularly in areas with lower cell density. This is a direct consequence of the standardized settings applied to maintain comparability and highlight the morphological features. The acquired imaging data, initially in TIFF format, was converted into IMS format via Imaris FileConverter (Andor Technologies, GB) and further analyzed with Imaris software (Andor Technologies, GB, RRID:SCR_007370). To quantify morphological differences in the GBM cultures, two distinct Surface algorithms were applied. Debris and round cells were excluded based on signal intensity and volume, while filamentous structures and spheroids were differentiated using sphericity values (filamentous structures: sphericity <0.5; spheroids: sphericity ≥0.5). 3D objects were generated for each identified structure within the z‐stacks, and CSV data files containing object properties were generated for each image. A custom MATLAB (MathWorks, US, RRID:SCR_001622) script, along with Microsoft Excel (Microsoft, US, RRID:SCR_016137) routines, was developed to merge and process the exported CSV files. This enabled quantification and visualization of the desired outputs: total filament volume, spheroid count per well, mean spheroid volume, and total spheroid volume. These metrics have previously been used to characterize invasion phenotypes in ECM‐supported co‐culture models [66]. Graphical representations of the data were generated using Prism 6 (GraphPad, US).

### 4.7 Statistical Analysis

Outliers were identified using the ROUT method (Q = 1%) in GraphPad Prism 6 (GraphPad Software, San Diego, CA, USA; RRID:SCR_002798). To assess the distribution of the data, normality was tested using both normality and lognormality tests provided by the software. Based on the results, parametric tests, including unpaired t‐tests for two‐group comparisons or one‐way ANOVA for multiple group comparisons, were applied to normally distributed data. For data that did not meet normality assumptions, appropriate non‐parametric tests were used. Box plots were generated to visualize the distribution of data, with the Tukey method applied to identify and highlight outliers. Multiplicity‐adjusted *p*‐values below 0.05 were considered statistically significant, where ^*^ *p* <0.05, ^**^ *p* <0.01, ^***^ *p* <0.001, and ^****^ *p* <0.0001.

### 4.8 RNA Extraction and Whole Transcriptome Sequencing

We selected LN229 for these bulk RNA‐seq comparisons because it is well characterized in the literature, grows reproducibly in our 3D hydrogel, and provides a stable baseline to illustrate material‐driven effects prior to expanding to more heterogeneous patient‐derived samples. Total RNA was extracted from the 3D encapsulated cells using the Norgen`s Total RNA purification kit, [Norgen Biotek Corporation], following the manufacturer's protocol. Some steps were optimized for the extraction process, as outlined in previous studies [67, 68]. For each drug condition‐ 5‐FU/U, carmustine, and their combination, RNA was isolated from 12 wells (biological replicates) of 3D cell cultures, including untreated controls to enhance data reliability. The RNA samples from each condition and controls were pooled and sequenced together. Briefly, cells were lysed in Buffer RL and homogenized to ensure complete cell disruption. RNA was isolated via column‐based purification, and on‐column DNase treatment was performed to remove any genomic DNA contamination. The RNA quality and concentration were assessed using a NanoDrop spectrophotometer (Thermo Fisher Scientific, US). Additionally, RNA integrity was first assessed using the Agilent 2100 Bioanalyzer (Agilent Technologies, US) with the Eukaryote Total RNA Pico Kit, providing RNA Integrity Numbers (RIN) to ensure high‐quality RNA (RIN ≥ 8.0) for sequencing. Also, 300 ng of total RNA per sample was analyzed using the Fragment Analyzer NGS LAB3877 (Agilent Technologies) to confirm the size distribution and integrity of the RNA, further ensuring suitability for downstream library preparation and sequencing. Polyadenylated RNA was enriched from the total RNA using oligo(dT) magnetic beads to isolate mRNA. For samples requiring depletion of ribosomal RNA, we used an rRNA depletion kit (e.g., NEBNext rRNA Depletion Kit, NEB). cDNA synthesis was performed using a reverse transcription kit (e.g., SuperScript IV, Thermo Fisher Scientific), followed by fragmentation and adapter ligation using the NEBNext Ultra II RNA Library Prep Kit (NEB). The prepared libraries were amplified using polymerase chain reaction (PCR), quantified with a fluorometric assay (e.g., Qubit, Thermo Fisher Scientific), and validated for fragment size distribution using an Agilent Bioanalyzer. Sequencing‐ready libraries were loaded onto an Illumina sequencing platform for paired‐end sequencing, generating 100‐bp reads. Following quality control checks on raw sequencing reads using FastQC, we aligned the reads to the reference genome (e.g., GRCh38 for human samples) using the STAR aligner, ensuring high mapping efficiency and accurate transcriptome quantification [67, 69]. Post‐alignment, gene‐level read counts were quantified using featureCounts, a robust tool for mapping sequencing reads to genomic features. To identify differentially expressed genes (DEGs), we used the DESeq2 package, which applies a negative binomial distribution model to account for biological variability between replicates. Genes with an adjusted *p*‐value < 0.05 were considered significantly differentially expressed. The DEGs were further analyzed for enriched biological pathways using Gene Ontology (GO) and Kyoto Encyclopedia of Genes and Genomes (KEGG) pathway enrichment analysis, performed through clusterProfiler.

### 4.9 Transcriptomic Comparison to Public GBM Datasets

Bulk RNA‐seq count data for primary and recurrent glioblastoma (GBM) specimens were obtained from the NCBI Gene Expression Omnibus (GEO) under accession GSE15824. This dataset comprises Illumina‐generated transcriptomes of primary (*n* = 15) and recurrent (*n* = 10) human GBM tissues. Raw FASTQ files were downloaded via the GEO FTP server; accompanying metadata (patient age, sex, treatment status) were retrieved from the GEO sample records. Raw reads were quality‐checked using FastQC v0.11.9 and trimmed for adapters and low‐quality bases (Phred < 20). Reads were aligned to the human reference genome (GRCh38.p13) using STAR v2.7.10a in 2‐pass mode. Gene‐level read counts were generated with featureCounts v2.0.4 against GENCODE v40 gene annotations. Transcript counts from GSE15824 and our in‐house LN229 RNA‐seq experiments were combined into a single matrix. Low‐expression genes (< 1 count per million in at least three samples) were removed. Counts were normalized by trimmed mean of M‐values (TMM) in edgeR v3.38.4, and log_2_‐transformed using voom in limma v3.52.2. To mitigate batch effects between public and in‐house datasets, we applied ComBat‐Seq from the sva package v3.44.0 with sample origin (GEO vs. in‐house) as the batch factor. We modeled gene expression differences between samples, fitting a design matrix that included culture condition and batch. Genes with an adjusted p‐value (Benjamini–Hochberg FDR) < 0.05 and |log_2_ fold‐change| > 1 were considered differentially expressed. To identify pathways enriched in comparison of different culture conditions and to link these to primary or recurrent GBM signatures, we performed Gene Ontology (GO) over‐representation analysis with clusterProfiler v4.6.0. We separately tested (i) genes up‐regulated in 3D cultures against the background of all expressed genes, and (ii) genes identified as significantly higher in primary or recurrent GBM (from GSE15824 differential analysis between primary vs. recurrent). GO terms with FDR < 0.05 were retained and grouped into functional categories (e.g., ECM organization, cell adhesion, protease activity). Heatmaps of selected GO terms and normalized expression values were generated using pheatmap v1.0.12. Volcano plots for differential expression and bar plots of top GO categories were created in ggplot2 v3.4.4.

## Author Contributions

R. Kaur‐Trautmann: conceptualization, project administration, methodology, investigation, visualization, data analysis, formal analysis writing – original draft, writing – review and editing. N. Dennison: methodology, investigation, data analysis, formal analysis. K. McCortney: resources, patient‐derived cell lines. S. Klier: investigation, data analysis. M.I. Cosacak: data analysis, formal analysis. C. Werner: resources, funding acquisition. G. Akyoldas: resources, patient‐derived cell lines. C.M. Horbinski: resources, patient‐derived cell lines, writing – review and editing. U. Freudenberg: supervision, funding acquisition, methodology. C. Kizil: conceptualization, supervision, funding acquisition, methodology, writing – original draft, writing – review and editing. All authors reviewed and approved the final manuscript.

## Conflicts of Interest

C.K. is a scientific advisor to Neuron D GmbH. The other authors declare no conflicts of interest.

## Supporting information




**Supporting File 1**: mabi70129‐sup‐0001‐SuppMat.docx.

**Supporting File 2**: mabi70129‐sup‐0002‐Figure S1.jpg.

**Supporting File 3**: mabi70129‐sup‐0003‐Figure S2.jpg.

**Supporting File 4**: mabi70129‐sup‐0004‐Figure S3.jpg.

**Supporting File 5**: mabi70129‐sup‐0005‐Figure S4.jpg.

**Supporting Information**: mabi70129‐sup‐0006‐Table S1.xlsx.

**Supporting File 6**: mabi70129‐sup‐0007‐DataFile.7z.

## Data Availability

The RNA sequencing data generated in this manuscript is deposited to NCBI's GEO (https://www.ncbi.nlm.nih.gov/geo) with accession number GSE290610. Other data generated in this study are available within the article and its supplementary data files.
