# Supplementary material for: High‐Throughput 3D Glioblastoma Model in Glycosaminoglycan Hydrogels for Personalized Therapeutic Screening
Source: Macromol Biosci. 2026 Jan 14;26(1):e00394. doi: 10.1002/mabi.202500394 (PMC12805317; doi:10.1002/mabi.202500394)
Supplement: Supplementary file 1 — Supporting File 1: mabi70129‐sup‐0001‐SuppMat.docx. [file MABI-26-e00394-s005.docx]

**
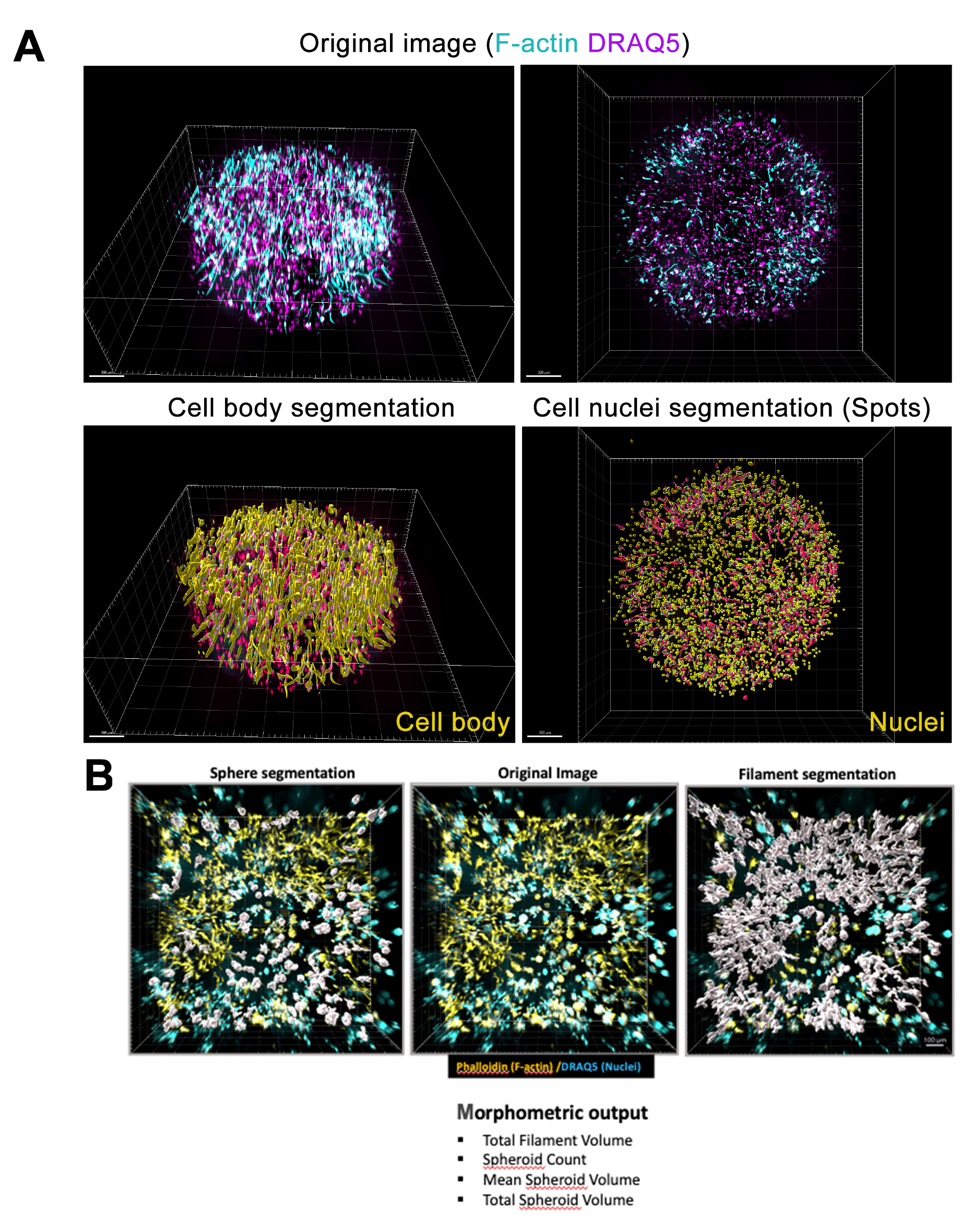
Supplementary Fig. 1: High-content 3D segmentation workflow and morphometric readouts.** **(A)** Raw image acquisition and segmentation of cellular components. Top panels show maximum-intensity projections of a representative LN229 filaments and spheroid in starPEG–GAG hydrogel, stained for F-actin (phalloidin; cyan) and nuclei (DRAQ5; magenta). Bottom left panel overlays the 3D “cell body” surface (yellow) generated by filament tracing in Imaris on the raw channels. Bottom right panel shows the corresponding nuclear “spot” detection (yellow) overlaid on the original image. Scale bars = 200 µm. **(B)** Automated identification of spheroids and invasive filaments. From left to right: (i) surfaces corresponding to compact spheroid bodies (white) segmented based on size and sphericity criteria; (ii) the original composite image; and (iii) filamentous structures (gray) extracted from the F-actin channel using a length-based filtering algorithm. The table summarizes the four primary morphometric outputs used for downstream analysis. Scale bars = 100 µm.


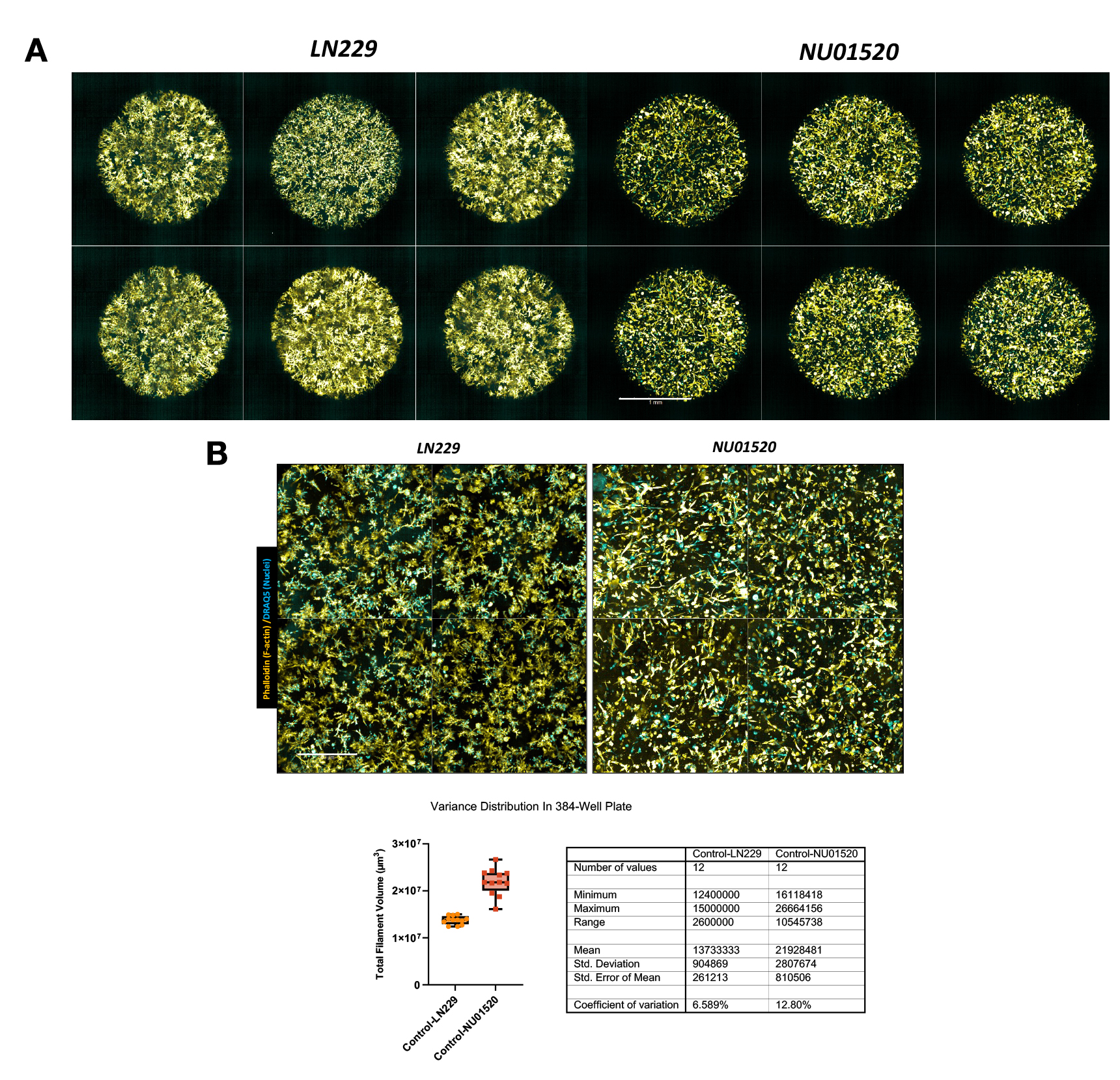
**Supplementary Fig. 2: Reproducibility and well‐to‐well variability of filamentous invasion across GBM lines.** **(A)** Representative maximum-intensity projections of phalloidin-stained F-actin (yellow) and DRAQ5-stained nuclei (cyan) from six independent wells each of LN229 (left six panels) and NU01520 (right six panels) cultured in starPEG–GAG hydrogels for 7 days. These images illustrate the consistent emergence of invasive filamentous and spheroids structures within the hydrogel across biological replicates. Scale bar = 1 mm. **(B)** Top: Four randomly selected fields per well (stitched 2×2 tiles; scale bar = 500 µm) for LN229 (left) and NU01520 (right) control wells, demonstrating uniform coverage and morphology across the 384-well plate. Bottom left: Box-and-whisker plot of total filament volume (µm³) measured in 12 control wells per cell line, showing tight distributions. Bottom right: Summary statistics for LN229 and NU01520 total filament volumes, including minimum, maximum, range, mean ± SD, standard error of the mean, and coefficient of variation (CV). The low CV values (< 13%) confirm high inter‐well reproducibility of the invasion readout.


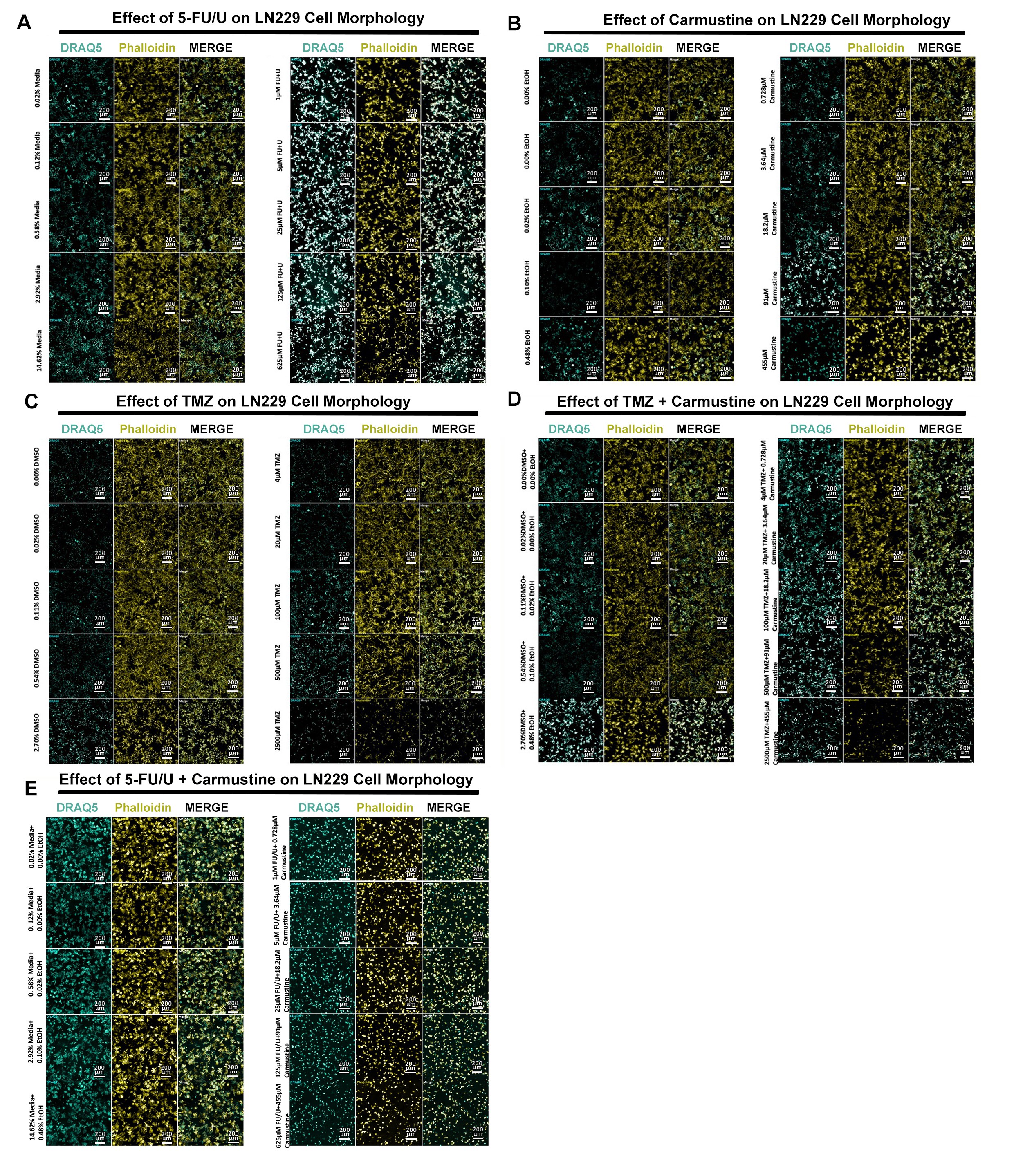


**Supplementary Fig. 3: Dose-dependent effects of monotherapies and combination treatments on LN229 invasive morphology in 3D GBM cultures.** Each panel shows a confocal projection of LN229 cells in starPEG–GAG hydrogels after 7 days of treatment, stained for F-actin (phalloidin; yellow) and nuclei (DRAQ5; cyan). Left subpanels in each row display increasing solvent or drug concentrations in the carrier control (media + vehicle), while right subpanels show matched concentrations of the active compound(s). All scale bars = 200 µm. **(A)** 5-Fluorouracil and uracil (5-FU/U) monotherapy: dose escalation from 1 µM to 625 µM reveals progressive reduction in filamentous structures. **(B)** Carmustine monotherapy: 0.728 µM to 455 µM showing cell rounding and reduces invasive protrusions only at higher doses of 455 µM **(C)** Temozolomide (TMZ) monotherapy: 4 µM to 2500 µM illustrates threshold-dependent effects on filament volume. **(D)** TMZ + carmustine combination: co-treatment with escalating TMZ (20–2500 µM) plus carmustine (3.64 µM to 455 µM) doses demonstrates synergistic suppression of invasion. **(E)** 5-FU/U + carmustine combination: co-treatment with 5-FU/U (1–625 µM) plus carmustine (0.728µM to 455 µM) at all tested doses nearly abolishes filaments, highlighting strong combinatorial efficacy.


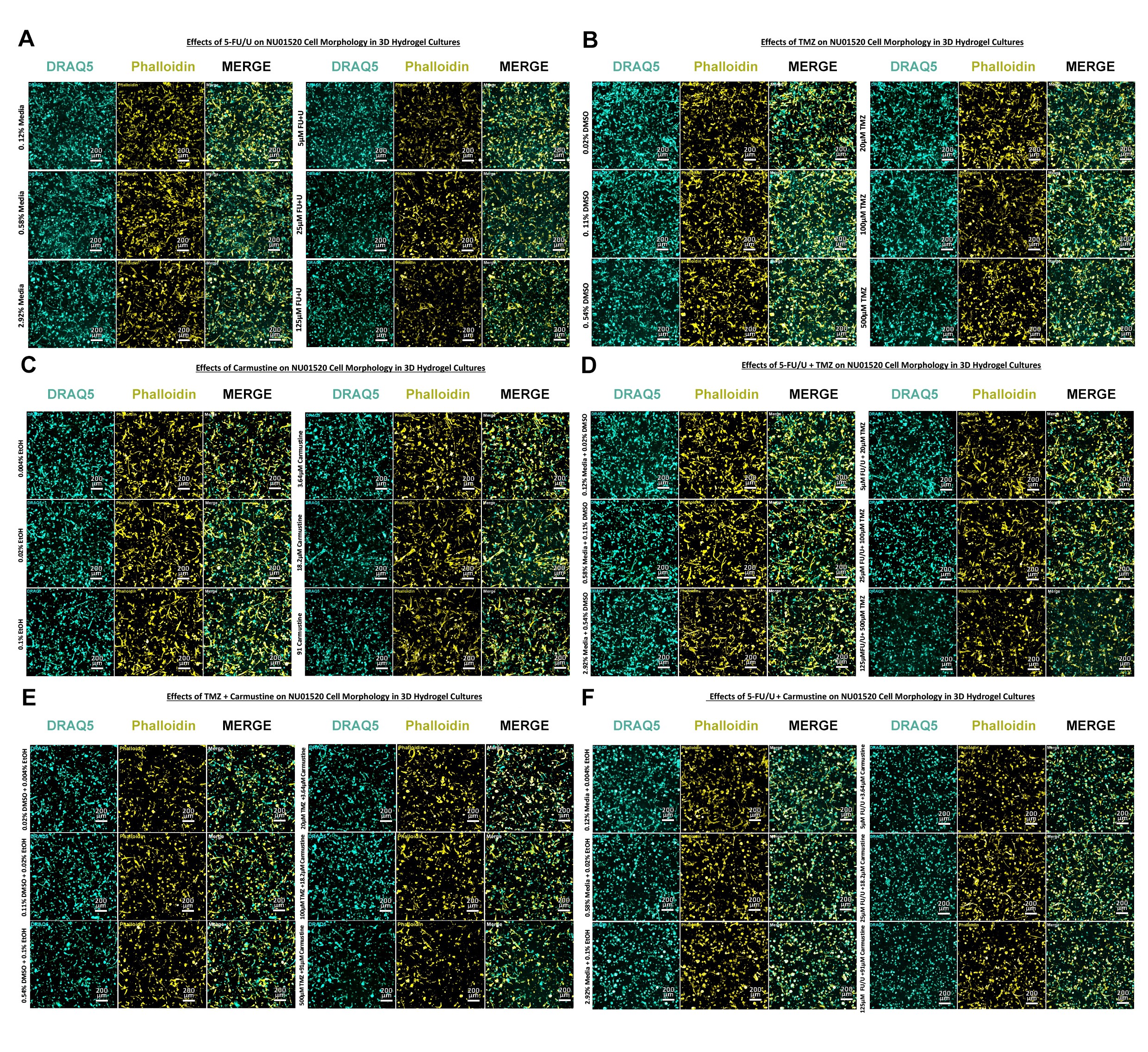


**Supplementary Fig. 4: Morphological responses of NU01520 cells to monotherapies and combinatorial treatments in 3D hydrogel cultures.** Each panel shows a confocal projection of NU01520 cells in starPEG–GAG hydrogels after 7 days of treatment, stained for F-actin (phalloidin; yellow) and nuclei (DRAQ5; cyan). Within each subpanel, the left column shows dose-matched vehicle controls (media, ethanol, DMSO, or their combinations), and the right column shows escalating concentrations of the active drug(s). All scale bars = 200 µm. **(A)** 5-Fluorouracil with uracil (5-FU/U) monotherapy (5 µM–125 µM) demonstrating graded reduction in filament formation. **(B)** Temozolomide (TMZ) monotherapy (20 µM–500 µM) showing no significant morphological changes across the tested dose range. **(C)** Carmustine monotherapy (3.64 µM–91 µM) showing no significant cell rounding or suppression of invasion. **(D)** 5-FU/U + TMZ combination (5–125 µM 5-FU/U + 20–500 µM TMZ) revealing additive effects on filament reduction at higher doses. **(E)** TMZ + carmustine combination (20–500 µM TMZ + 3.64 µM–91 µM carmustine) showing no significant changes at cell morphology **(F)** 5-FU/U + carmustine combination (5–125 µM 5-FU/U + 3.64 µM–91 µM carmustine) nearly abolishing filamentous extensions even at low 5-FU/U concentrations.

**Table S1: Source data (provided as an Excel file).** The supplementary sheet contains all raw values obtained from individual experiments, which were subsequently used to generate the graphs presented in the manuscript. The file comprises raw data arranged in separate tabs for each experiment, containing the individual measurements (e.g., filament volume, spheroid count, mean and total spheroid volume) for all treatment conditions, including controls and dose-dependent treatments for both the LN229 and the patient-derived NU01520 cell line.
