# Supplementary figures and images for: High‐Throughput 3D Glioblastoma Model in Glycosaminoglycan Hydrogels for Personalized Therapeutic Screening
Source: Macromol Biosci. 2026 Jan 14;26(1):e00394. doi: 10.1002/mabi.202500394 (PMC12805317; doi:10.1002/mabi.202500394)

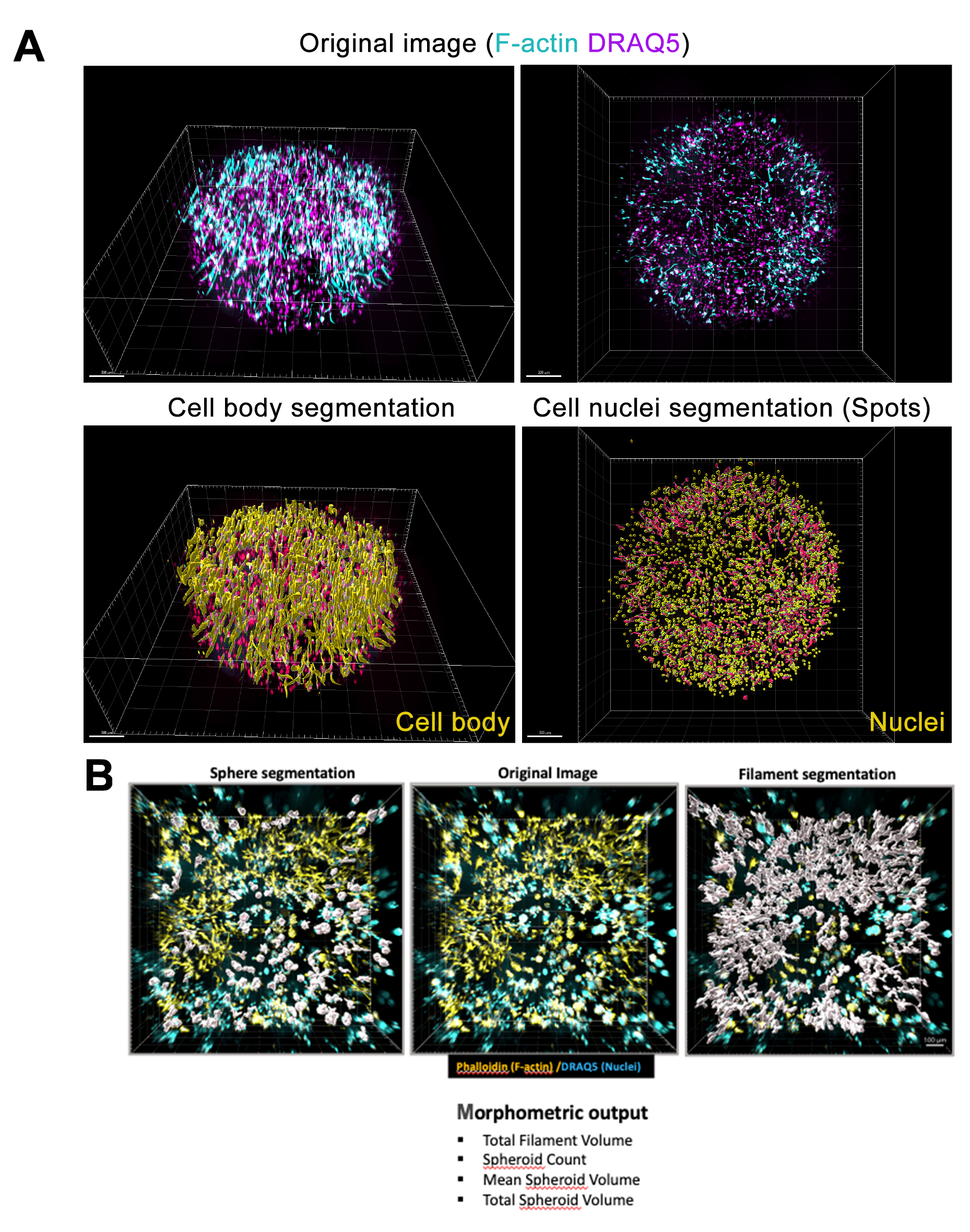

Supplement: Supplementary file 2 — Supporting File 2: mabi70129‐sup‐0002‐Figure S1.jpg. [file MABI-26-e00394-s007.jpg]

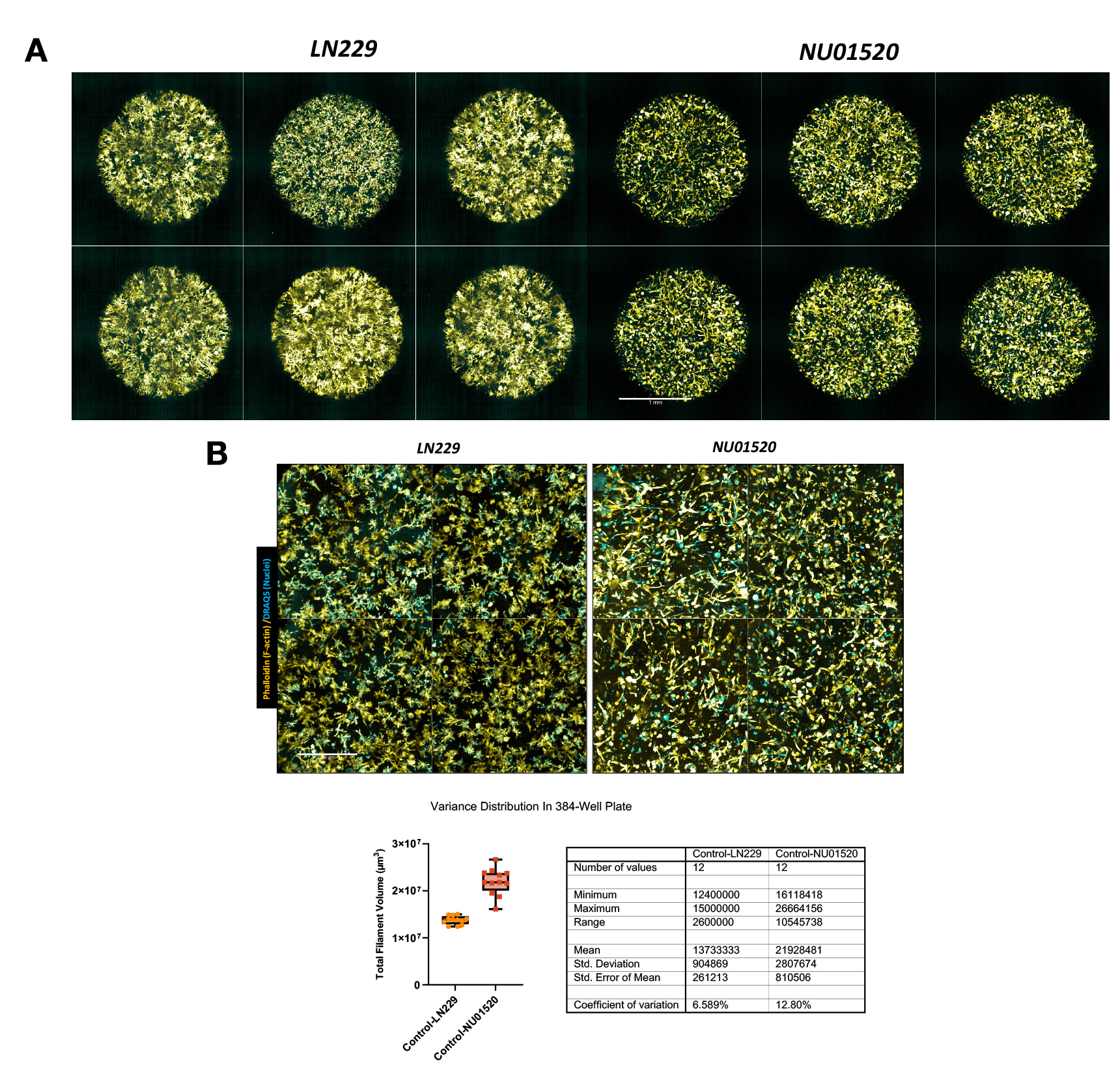

Supplement: Supplementary file 3 — Supporting File 3: mabi70129‐sup‐0003‐Figure S2.jpg. [file MABI-26-e00394-s004.jpg]

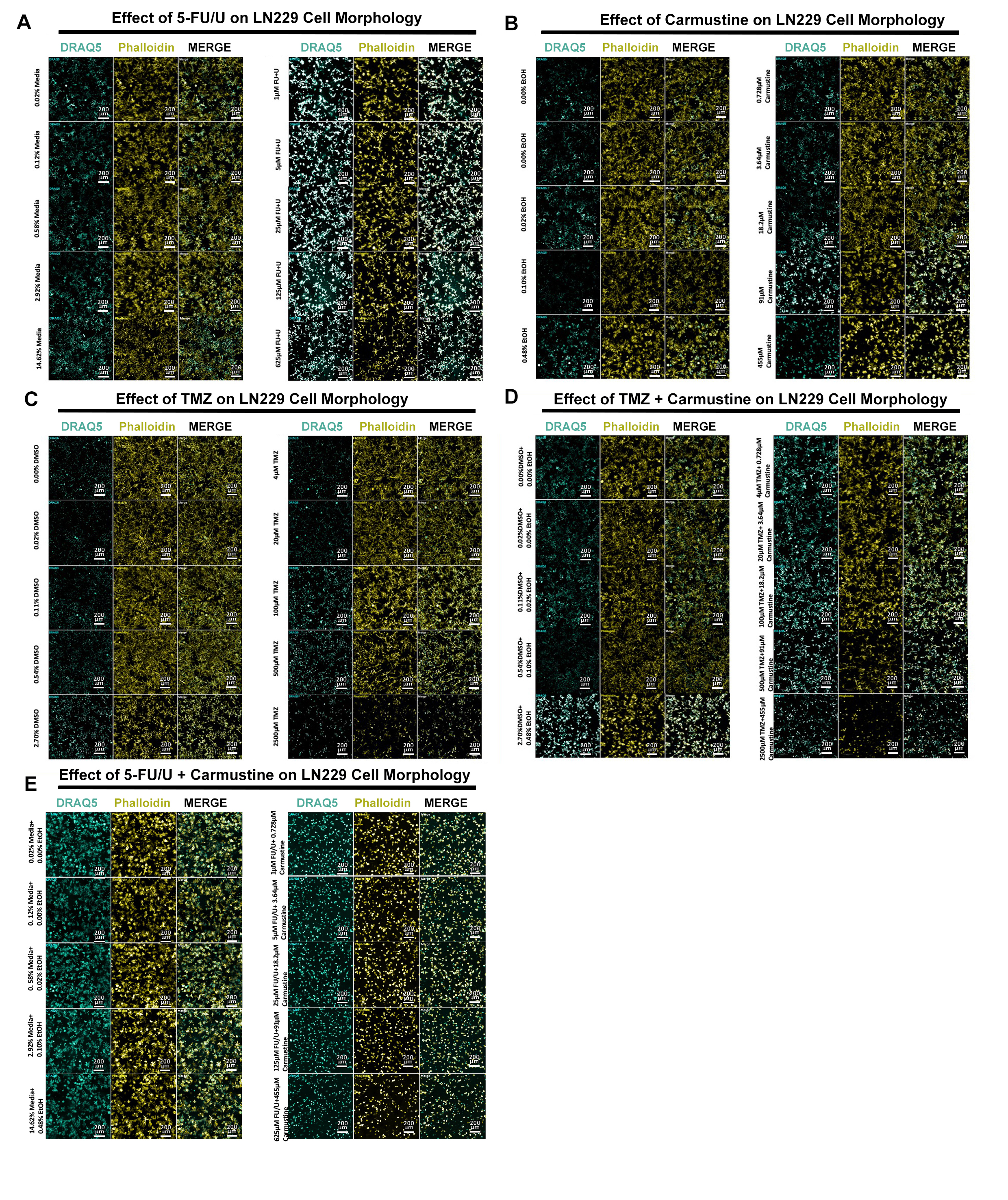

Supplement: Supplementary file 4 — Supporting File 4: mabi70129‐sup‐0004‐Figure S3.jpg. [file MABI-26-e00394-s001.jpg]

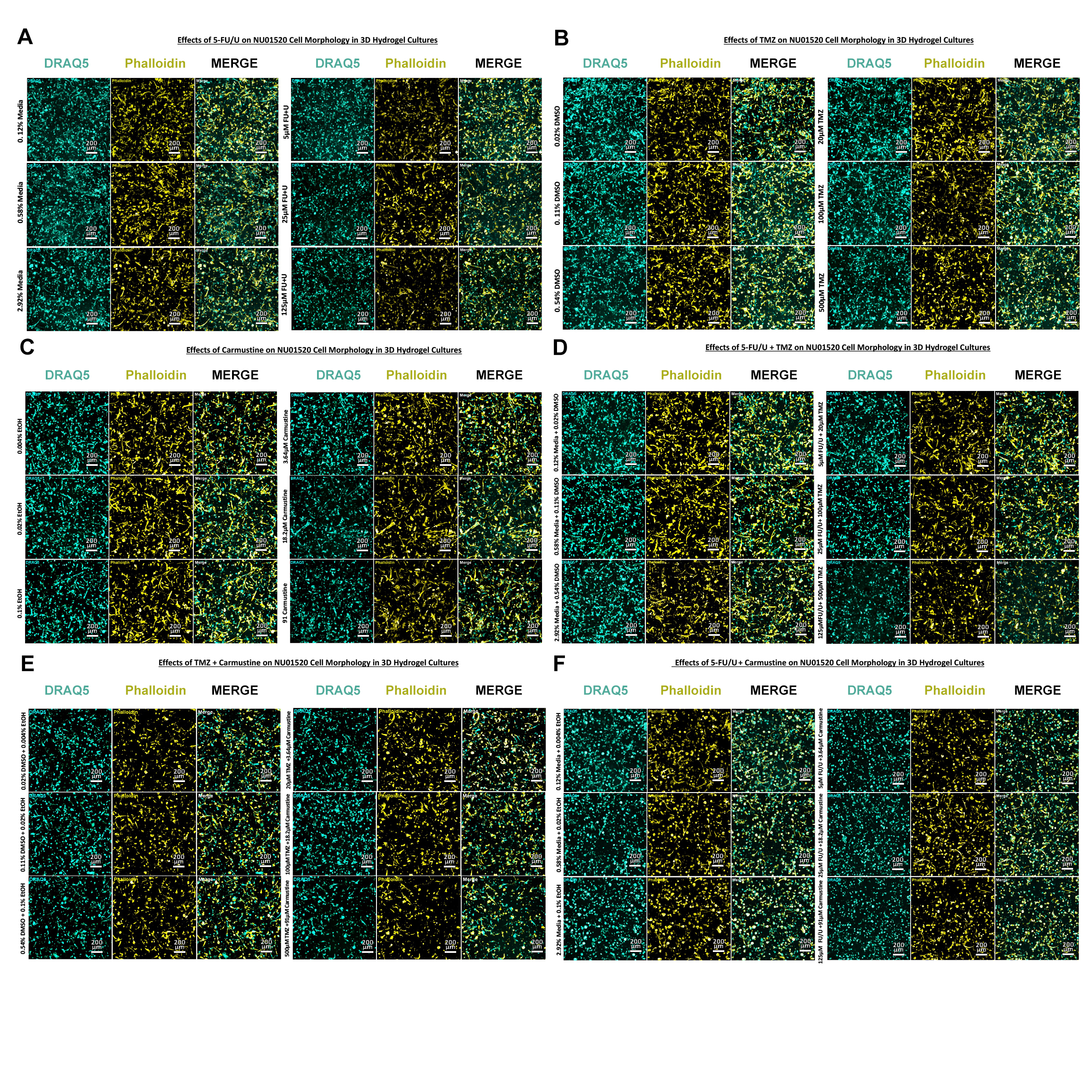

Supplement: Supplementary file 5 — Supporting File 5: mabi70129‐sup‐0005‐Figure S4.jpg. [file MABI-26-e00394-s002.jpg]
